# Tumefactive demyelinating lesions: a retrospective cohort study in Thailand

**DOI:** 10.1038/s41598-024-52048-w

**Published:** 2024-01-16

**Authors:** Tatchaporn Ongphichetmetha, Saharat Aungsumart, Sasitorn Siritho, Metha Apiwattanakul, Jantima Tanboon, Natthapon Rattanathamsakul, Naraporn Prayoonwiwat, Jiraporn Jitprapaikulsan

**Affiliations:** 1 Division of Neurology, Department of Medicine, Faculty of Medicine Siriraj Hospital, Mahidol University, 2 Wanglang Rd, Siriraj, Bangkok-noi, Bangkok, 10700 Thailand; 2 Siriraj Neuroimmunology Center, Faculty of Medicine Siriraj Hospital, Mahidol University, Bangkok, 10700 Thailand; 3 Division of Clinical Epidemiology, Department of Research and Development, Faculty of Medicine Siriraj Hospital, Mahidol University, Bangkok, 10700 Thailand; 4 Neuroimmunology Unit, Department of Neurology, Neurological Institute of Thailand, Bangkok, 10400 Thailand; 5 Bumrungrad International Hospital, Bangkok, 10110 Thailand; 6 Department of Pathology, Faculty of Medicine Siriraj Hospital, Mahidol University, Bangkok, 10700 Thailand

**Keywords:** Diseases of the nervous system, Multiple sclerosis, Neuroscience, Neurology, Neurological disorders, Demyelinating diseases, Multiple sclerosis

## Abstract

Tumefactive demyelinating lesions (TDL), characterized by large (≥ 2 cm) demyelinating lesions mimicking tumors, are a rare manifestation of the central nervous system inflammatory demyelinating diseases (CNS-IDD). Distinguishing TDL from other brain lesions can be challenging, often necessitating biopsy or advanced diagnostics. The natural history of TDL varies among races. This study aimed to assess demographics, clinical and radiological features, laboratory findings, management, and outcomes of Thai patients with TDL. We retrospectively reviewed records of twenty-six patients with TDL from the Multiple Sclerosis and Related Disorders registry from two tertiary medical centers. Among 1102 CNS-IDD patients, 26 (2.4%) had TDL. The median age at TDLs onset was 34.5 years (range 17–75); 69.2% were female. Over 70% manifested TDL as their initial CNS-IDD presentation. Common presenting symptoms included motor deficits, sensory disturbances, and cognitive problems. About two-fifths exhibited multiple lesions, most frequently in the frontoparietal region (46.2%). Half of the patients showed an incomplete ring on post-contrast T1-weighted imaging, with peripheral diffusion-weighted imaging restriction in twenty-one patients. T2-hypointense rims were present in thirteen (56.5%) patients. Brain biopsy was performed in 12 cases (46.1%). Serum aquaporin-4 immunoglobulin was positive in 16.7% of tested (4/24) cases. Serum myelin oligodendrocyte glycoprotein immunoglobulin was negative in all thirteen patients tested. Twenty patients (76.9%) received intravenous corticosteroids for TDL attacks. After the median follow-up period of 48 months (range 6–300), 23.1% experienced CNS-IDD relapses. Median Expanded Disability Status Scale at TDL diagnosis was 4.3 (range 0.0–9.5), and improved to 3.0 (range 0.0–10.0) at the last follow-up. This study suggested that TDL were rare among Thai CNS-IDD patients, frequently presenting as a monophasic condition with a favorable outcome.

## Introduction

Tumefactive demyelinating lesions (TDL), also known as tumor-like demyelinating lesions or pseudotumoral demyelinating lesions, constitute a unique neuropathological entity within the field of neurological disorders^1,2^. These lesions are characterized by large, inflammatory, demyelinating lesions, typically exceeding 2 cm in diameter. TDL were originally associated with presentations in patients with multiple sclerosis^3^. However, various etiologies underlying TDL formation have been identified, encompassing conditions such as acute disseminated encephalomyelitis^4,5^, neuromyelitis optica spectrum disorder (NMOSD)^6,7^, myelin oligodendrocyte glycoprotein antibody-associated disease (MOGAD)^8^, MS variants (namely Baló concentric sclerosis^9,10^, myelinoclastic diffuse sclerosis or Schilder’s disease^11^, and Marburg’s acute MS^12^), and autoimmune neurological disorders such as Behçet disease^13^ and neurosarcoidosis^14^. Moreover, TDL may result from infectious processes^15,16^, certain medications^17,18^, or occur in isolation, devoid of concomitant demyelinating pathologies.

Epidemiological studies of TDL have predominantly focused on MS patients’ cohorts. Recent population-based study, however, has illuminated the occurrence of TDL, revealing that 1.9% of individuals with MS have experienced TDL in the course of disease^19^. In a comprehensive retrospective cohort analysis, TDL exhibited a higher prevalence among MOGAD patients (22%) compared to NMOSD with aquaporin-4 antibodies (AQP4-IgG) (5%)^20^. Clinical manifestations of TDL exhibit substantial variability depending on their anatomical localization and the extent of perilesional involvement. Given the mass-like radiological characteristics and the unusual clinical manifestations associated with inflammatory demyelinating diseases of the central nervous system (CNS-IDD), it is essential to rigorously distinguish TDL from more common conditions such as primary brain tumors, brain metastases, brain abscess, other central nervous system infections, and primary CNS lymphoma before concluding a diagnosis^21^.

Previous research aimed at elucidating the radiological characteristics of TDL has revealed distinctive brain magnetic resonance imaging (MRI) features, including the presence of a T2-weighted (T2W) hypointense rim, relatively mild perilesional edema, a discernible mass effect, a central vein sign, or an appearance of an open ring upon gadolinium enhancement^22^. Pathological diagnosis is warranted in some cases. The complexities involved in diagnosing TDL can lead to delays in appropriate investigation and treatment. Moreover, morbidity associated with extensive investigations has been observed to be higher in TDL patients compared to those with typical MS. For instance, brain biopsy in TDL patients resulted in post-operative seizures or post-operative infections^23^. Enhancing the diagnostic process to circumvent the need for brain biopsy could lead to improved morbidity outcomes and facilitate the prompt initiation of definitive treatment.

Regarding prognosis, Lucchinetti et al. reported that 70% of 168 pathological confirmed patients with TDL from the USA and Germany developed clinically definite MS during a median follow-up period of 3.9 years^24^. On the other hand, a retrospective cohort study in China found that only 28% (33 out of 116 patients) with TDL met the diagnostic criteria for MS after a follow-up period of 72 months^25^. These findings highlight the potential variations in prognosis across different ethnicities.

Therefore, it is crucial to enhance recognition of the diverse clinical presentations, radiological characteristics, ancillary investigative modalities, clinical courses, and definitive diagnoses across diverse ethnic cohorts with TDL. The current study aimed to determine the prevalence of TDL within the CNS-IDD cohort in Thailand and elucidate the distinctive characteristics displayed by Thai TDL patients.

## Methods

This retrospective cohort study was conducted at two tertiary hospitals in Bangkok, Thailand: Siriraj Hospital and the Neurological Institute of Thailand. The study focused on patients with TDL, defined as brain lesions with a minimum of 2 cm in diameter, showing hyperintensity on T2-weighted images and mass-like appearances. Data were collected from Siriraj CNS-IDD registry and Neurological Institute of Thailand database, spanning from January 2015 to March 2023. Inclusion criteria were patients with TDL in any time point of their CNS-IDD disease course, with a minimum 6-month follow-up period. Patients diagnosed with non-inflammatory diseases such as brain tumors or primary CNS lymphoma were excluded. The study was approved by Siriraj Institutional Review Board (COA no. Si 391/2023) and the Institutional Review Board of the Neurological Institute of Thailand (approval number 66037) and all methods were performed in accordance with the guidelines and regulations. Informed consent was obtained from all participants for the use of de-identified medical records.

### Data collection

#### Demographic and clinical information

Demographic details, such as gender, comorbidities, prior diagnoses of CNS-IDD, and previous neurological status, were reviewed. Clinical presentations were recorded and categorized into neurological presentation domains (motor, sensory, visual, brainstem, consciousness, cognition, headache, seizures, movement, bowel and bladder). These domains are defined in Supplementary materials. We determined whether these manifestations could be attributed to a single anatomical region (monofocal) or involved multiple anatomical regions (polyfocal). Additionally, the duration from symptom onset to the first hospital visit, the duration from the initial hospital visit to the TDL diagnosis, and any identified risk factors associated with CNS-IDD were collected.

#### Radiological data

**Figure 1:**
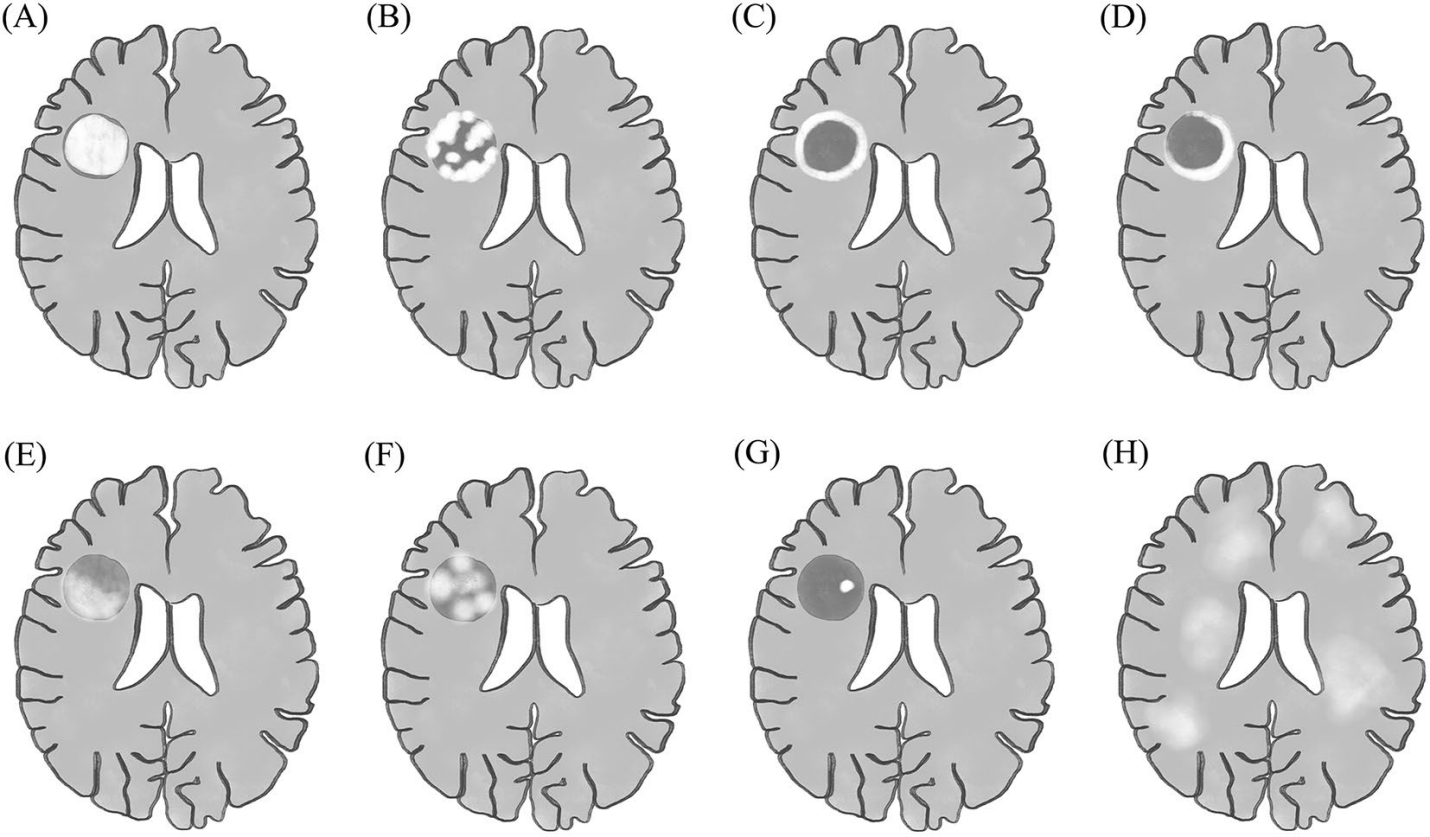
illustrates typical gadolinium-enhancing patterns of tumefactive demyelinating lesions (TDL), including (**A**) homogenous (dense and uniform enhancement across the entire lesion) (**B**) heterogenous (inconsistent and complicated pattern and arrangement of enhancement) (**C**) closed ring (a complete circular enhancing border) and (**D**) open ring (an incomplete enhancing border, with the open portion extending into the gray matter). In selected TDLs, the heterogenous pattern could be further specified as (**E**) patchy (irregular and discontinuous areas of enhancement within a specific lesion) (**F**) nodular (clearly delineated areas of enhancement, each measuring > 2 mm, within regions that do not exhibit enhancement) (**G**) punctate (clearly delineated areas of enhancement, each measuring < 2 mm, within regions that do not exhibit enhancement) or (**H**) cotton-ball (an appearance of small, round, and clustered areas of enhancement).

Brain MRI and computer tomography (CT) scans underwent comprehensive evaluation in collaboration with neuroradiologists. We documented the duration from the onset of symptoms to the scan. Complete radiological findings were gathered, encompassing lesion number, location, size, gadolinium enhancement pattern (homogeneous, heterogeneous, closed ring, open ring, patchy, or nodular, as depicted in Fig. 1), presence of restricted diffusion on diffusion-weighted imaging (DWI), the extent of perilesional edema (mild [< 1 cm from the lesion], moderate [1 to 3 cm from the lesion], or marked [> 3 cm from the lesion])^24^, and mass effect (mild [sulcal effacement], moderate [< 1 cm subfalcine or uncal herniation], or marked [> 1 cm subfalcine or uncal herniation])^24^. Furthermore, we noted the presence of established typical TDL features, including the T2W hypointense rim (a noticeable, complete, thin border of T2W hypointensity contrasting with the hyperintense regions of the lesion's core and the surrounding edema) and the central vein sign (a hypointense thin line or small dot visible in at least two planes, centrally situated within the lesion, seen on susceptibility- or T2*-weighted imaging).

Details of TDL characteristics observed on brain CT scans were also recorded. Besides, in certain cases where data were available, we recorded findings from perfusion-weighted imaging (MR Perfusion) and Magnetic Resonance Spectroscopy (MRS).

#### Pathological data

Histopathological data for TDL were accessible in certain cases. We documented the duration from symptom onset to biopsy. The neuropathologist conducted a thorough review of the pathological findings.

#### Other ancillary investigations

All patients in the study underwent lumbar puncture. Cerebrospinal fluid (CSF) was routinely analyzed for white cell counts, protein levels, and glucose concentrations. CSF cytology and oligoclonal bands (OCBs) were available in some cases. Additional investigations encompassed the assessment of serum and CSF AQP4-IgG (using an in-house cell-based assay [CBA] in 2009–2010 and a commercial CBA from Euroimmun® thereafter), serum and CSF myelin oligodendrocyte glycoprotein antibodies (MOG-IgG) (using an in-house fixed CBA), other systemic autoantibodies, ophthalmic examinations, and spinal MRI.

#### Treatment and prognosis

Acute management of TDL includes, but is not limited to, intravenous corticosteroids, therapeutic plasma exchange, intravenous immunoglobulin, and prophylactic decompressive craniectomy. We documented the types, duration, and number of cycles of specific treatment for acute TDL attacks. Furthermore, we recorded details regarding specific maintenance therapy, such as an immunosuppressive agent or a disease-modifying therapy.

All patients were followed up for at least 6 months following the diagnosis of TDL. Prognostic data included the Expanded Disability Status Scale (EDSS) scores^26^ at diagnosis and at the last follow-up, the change or the resolution of lesions on follow-up MRI scans when available, and the occurrence of subsequent CNS-IDD attacks with time elapsed since the TDL attack. The total follow-up time, the cumulative number of TDL attacks, the total number of CNS-IDD attacks, and CNS-IDD clinical course (monophasic or relapsing–remitting) were summarized. The final diagnosis was ultimately determined for each patient.

### Statistical analysis

Continuous variables were presented as either the mean with standard deviation (SD) for normally distributed data or the median with range for non-normally distributed data. Categorical variables were expressed as percentages. The threshold for statistical significance was set as *p*-value of less than 0.05.

Statistical analyses and data processing were conducted using PASW Statistics for Windows version 18.0 (SPSS Inc., Chicago, IL, USA) and PRISM version 9.0 (GraphPad, San Diego, CA, USA).

## Results

Out of a total of 1102 patients with CNS-IDD, TDL were diagnosed in 26 individuals, constituting 2.4% of all cases from Siriraj CNS-IDD registry and Neurological Institute of Thailand database^27^. The median age at onset was 34.5 years (range 17–75) (Table 1). Among these, 18 (69.2%) were female, and 6 (23.1%) had previous CNS-IDD diagnoses, including 3 cases of acute myelitis, 2 cases of optic neuritis, and 1 case of NMOSD with AQP4-IgG. Interestingly, only 5 patients (19.2%) reported recent infections or exposure to vaccination within one month prior to the TDL diagnosis.

**Table 1:** Demographic characteristics of 26 patients within the cohort.

Demographic characteristics	N = 26
Female sex, n (%)	18 (69.2)
Age at TDLs onset, years^¶^	34.5 (17–75)
Previous CNS-IDD diagnosis, n (%)	6 (23.1)
Idiopathic acute myelitis	3
Idiopathic optic neuritis	2
NMOSD with AQP4-IgG	1
Presence of co-morbidities*, n (%)	7 (26.9)
Risk factors of CNS-IDD, n (%)	5 (19.2)
Recent infections within 1 month	4
Recent vaccination within 1 month	1

AQP4-IgG, aquaporin-4 antibody; CNS-IDD, inflammatory demyelinating disease of the central nervous system; NMOSD, neuromyelitis optica spectrum disorder; TDL, tumefactive demyelinating lesion.

^¶^Quantitative variables are displayed as the median (range).

*Details of co-morbidities are shown in Table S1 in Supplementary materials.

### Clinical information

**Figure 2:**
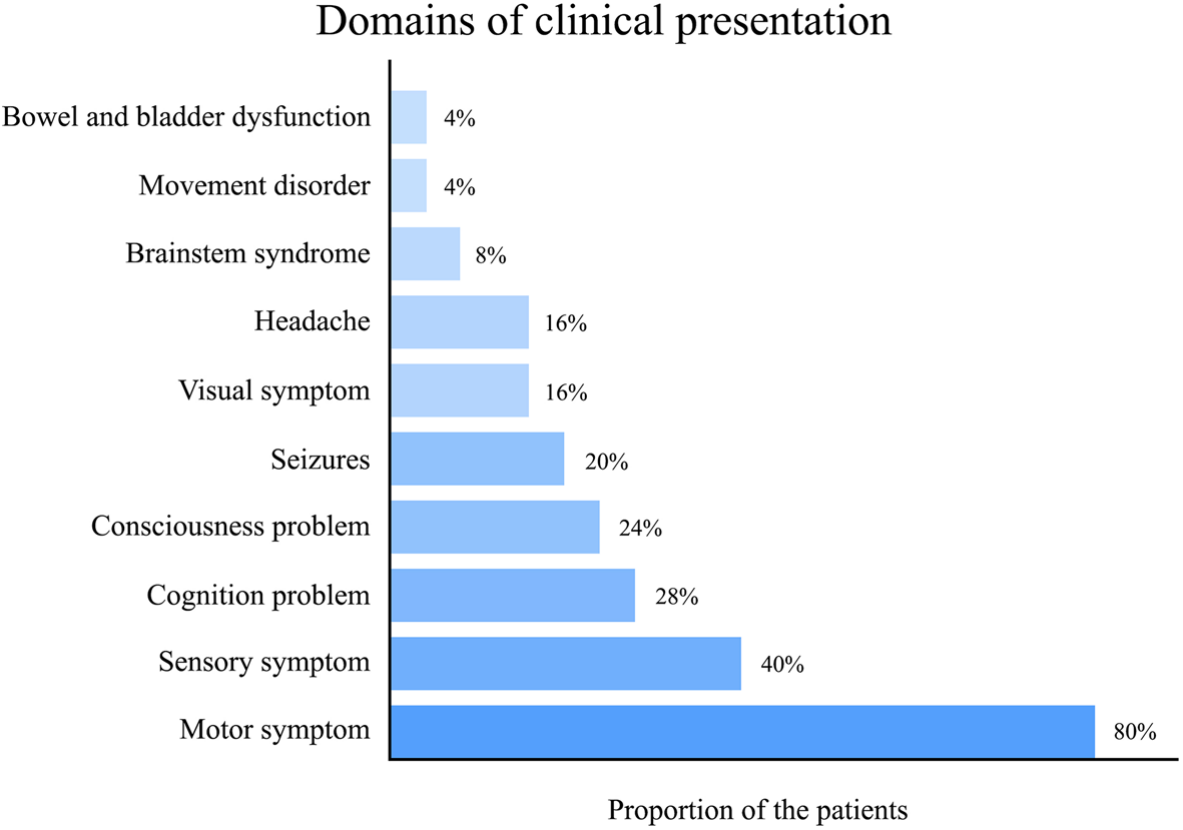
Frequency of each domain in the clinical presentation of tumefactive demyelinating lesions during the index attack.

Apart from one asymptomatic patient in this cohort (patient 12 in Supplementary materials), information regarding the clinical manifestations of the remaining 25 cases was summarized in Table 2. The most predominant domains of clinical presentation were motor (80%), followed by sensory symptoms (40%), and cognitive deficits (28%), as depicted in Fig. 2. Notably, the clinical presentations in the majority of patients were polysymptomatic (76.9%), but clinical manifestations were monofocal, indicative of involvement in a specific anatomical region, in seventeen patients (65.4%). The clinical course for nearly all patients (92%) was subacute, spanning from one week to less than three months. Furthermore, the median duration from the initial hospital visit to definitie diagnosis was 39 days (range 2–293).

**Table 2:** Clinical presentations of the 25 symptomatic patients in the cohort*

Clinical presentations	Symptomatic patients (n = 25)*
Domains of clinical presentations, n (%)	
Motor symptom	20 (80)
Sensory symptom	10 (40)
Cognitive problem	7 (28)
Consciousness problem	6 (24)
Seizures	5 (20)
Visual symptom	4 (16)
Headache	4 (16)
Brainstem syndrome	2 (8)
Movement disorder	1 (4)
Bowel and bladder dysfunction	1 (4)
Anatomical involvement from the clinical presentations, n (%)	
Monofocal	17 (68)
Polyfocal	8 (32)
Clinical course (time from onset to nadir), n (%)	
Subacute (1 week to < 3 months)	23 (92)
Chronic (≥ 3 months)	2 (8)
Time from symptom onset to first hospital visit^¶^, days	10 (0–180)
Time from first hospital visit to diagnosis^¶^, days	39 (2–293)

*One patient (patient 12 in Supplementary materials) was asymptomatic, hence the analysis of clinical information was based on the remaining 25 patients.

^¶^Quantitative variables are displayed as the median (range).

### Radiological data

**Figure 3:**
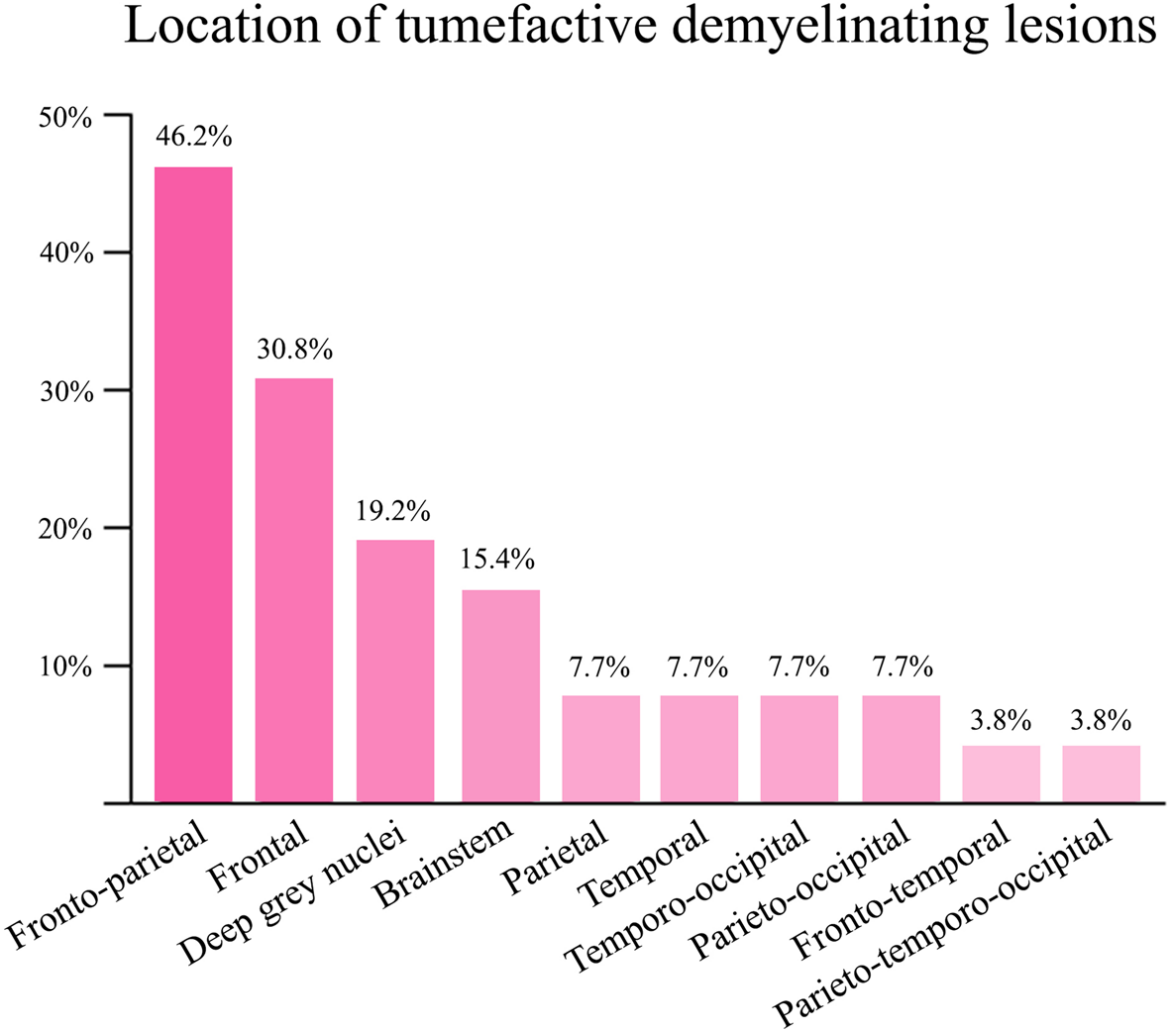
Frequency of anatomic locations of tumefactive demyelinating lesions.

Brain MRI finding analysis was presented in Table 3. Sixteen patients (61.5%) presented with solitary lesions. The distribution of TDL locations in the study cohort is depicted in Fig. 3. The most prevalent TDL locations were the fronto-parietal region (46.2%) and the frontal region (30.8%). Deep grey nuclei, including the thalamus and basal ganglia, were also affected in 19.2%. Eighteen patients (69.2%) had TDL with a maximal diameter of less than 5 cm.

**Table 3:** Brain magnetic resonance imaging characteristics of 26 patients in the cohort.

Characteristics	N = 26
Time from symptom onset to MRI*^,¶^, days	30 (6–180)
Numbers of TDL^¶^	1 (1–10)
TDL focality, n (%)	
Solitary lesion	16 (61.5)
Multiple lesions (≥ 2 lesions)	10 (38.5)
TDL laterality, n (%)	
Right	10 (38.4)
Left	8 (30.8)
Bilateral	8 (30.8)
TDL locations, n (%)	
Fronto-parietal	12 (46.2)
Frontal	8 (30.8)
Deep grey nuclei	5 (19.2)
Brainstem	4 (15.4)
Parietal	2 (7.7)
Temporal	2 (7.7)
Temporo-occipital	2 (7.7)
Parieto-occipital	2 (7.7)
Fronto-temporal	1 (3.8)
Parieto-temporo-occipital	1 (3.8)
TDL maximal diameter^€^, n (%)	
< 5 cm	18 (69.2)
≥ 5 cm	8 (30.8)
TDL volume^¶^, cm^3^	43.5 (6.6–280.0)
Patterns of gadolinium enhancement, n (%)	
Open ring	13 (50)
Heterogenous	5 (19.2)
Patchy	4 (15.4)
Homogenous	2 (7.7)
Closed ring	1 (3.8)
Nodular	1 (3.8)
Restriction on DWI^†^, n (%)	
Peripherally	21 (87.5)
Centrally	1 (4.2)
None	2 (8.3)
Perilesional edema^€^, n (%)	
Mild	21 (80.8)
Moderate	5 (19.2)
Marked	0 (0.0)
Mass effect^€^, n (%)	
Mild	20 (76.9)
Moderate	6 (23.1)
Marked	0 (0.0)
Central vein sign^ǂ^, n (%)	
Present	9 (45.0)
Absent	11 (55.0)
T2W hypointense rim^§^, n (%)	
Present	13 (56.5)
Absent	10 (43.5)
Involvement of the corpus callosum, n (%)	5 (19.2)
Other co-existing demyelinating lesions, n (%)	10 (38.5)
Periventricular	5 (19.2)
Brainstem	3 (11.5)
Optic nerve	3 (11.5)
Deep grey nuclei	2 (7.7)
Cortical or juxtacortical	1 (3.8)

DWI, diffusion-weighted imaging; MRI, magnetic resonance imaging; T2W, T2-weighted imaging; TDL, tumefactive demyelinating lesion.

^¶^Quantitative variables are displayed as the median (range).

^†^Diffusion-weighted imaging was available in 24 patients.

^€^According to the study by Lucchinetti et al.^24^.

^ǂ^T2*-weighted imaging and susceptibility-weighted imaging were available in 20 patients.

^§^T2W hypointense rim was evaluated in 23 patients.

**Figure 4:**
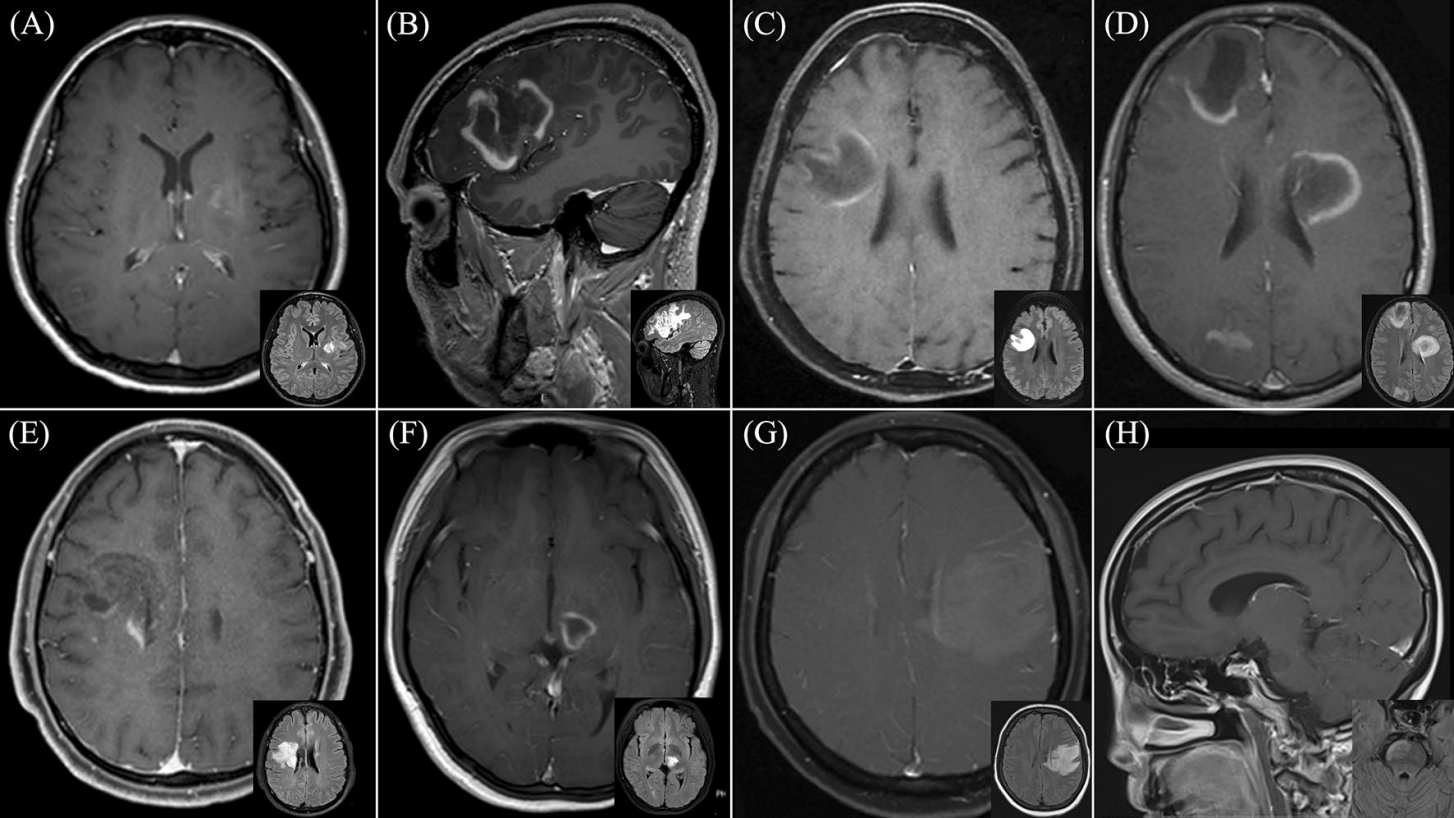
Representative brain magnetic resonance imaging (MRI) in six patients diagnosed with tumefactive demyelinating lesions (TDL). Post-gadolinium T1-weighted imaging is displayed in the larger picture, with a smaller Fluid-Attenuated Inversion Recovery (FLAIR) MRI provided in the lower right corner. (**A**) Patient 1: Heterogeneous enhancing pattern of the left basal ganglia lesion. (**B**) Patient 2: Open ring pattern of the left frontotemporal lesion. (**C**) Patient 12: Open ring pattern of the right frontoparietal lesion. (**D**) Patient 17: Open ring pattern of three lesions at the right frontal, left frontoparietal, right parietooccipital region. (**E**) Patient 20: Heterogeneous enhancing pattern of the right frontal lesion with Balo-like appearance. (**F**) Patient 22: Open ring pattern of the left thalamic lesion. (**G**) Patient 23: Patchy enhancing pattern of the left frontoparietal lesion. (**H**) Patient 25: Open ring pattern of the right-sided pontine lesion.

**Figure 5:**
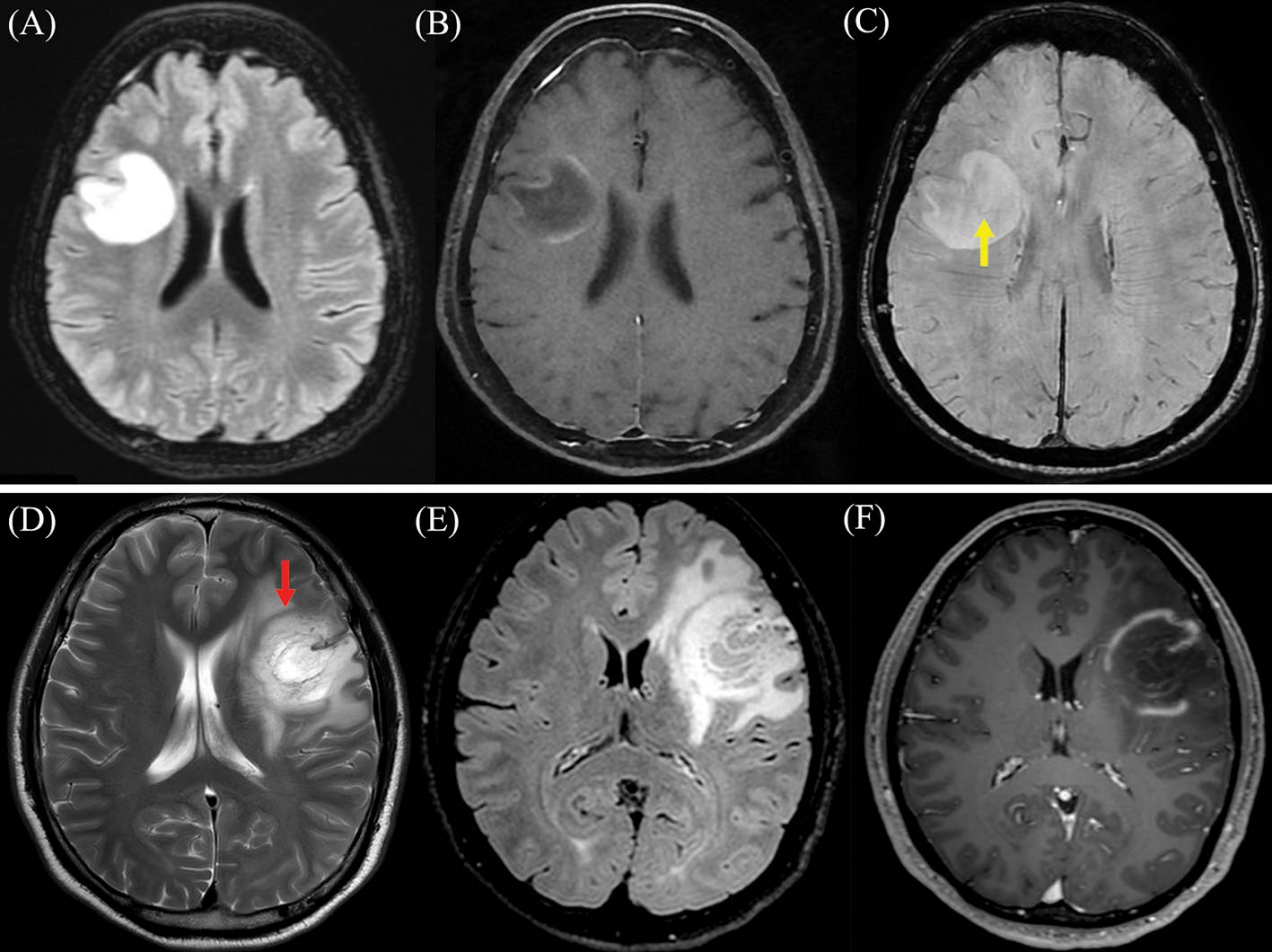
Representative brain magnetic resonance imaging (MRI) Patient 12: (**A**) Axial T2-weight Fluid-Attenuated Inversion Recovery (FLAIR), (**B**) Axial Post-gadolinium T1-weighted imaging, and (**C**) Axial T2 star-weighted angiography (SWAN) reveal the presence of the central vein sign (CVS), denoted by the yellow arrow. The CVS is characterized by a hypointense thin line or small dot centrally situated within the lesion. Patient 2: (**D**) Axial T2-weight imaging, (**E**) Axial T2-weight FLAIR, and (**F**) Axial Post-gadolinium T1-weighted imaging exhibit the T2-weighted hypointense rim, highlighted by the red arrow. The T2W hypointense rim is defined by a thin border of T2-weighted hypointensity, contrasting with the hyperintense regions of the lesion's core and the surrounding edema.

All TDL in the study demonstrated gadolinium enhancement, with open ring enhancement observed in half of the patients (50%). Upon reviewing diffusion-weighted imaging sequences, peripheral restricted diffusion, in which the area of restricted diffusion was at the lesion border, was observed in twenty-one patients (87.5%). Remarkably, we observed that the restricted diffusion area at the lesion border was typically the same area that showed gadolinium enhancement. Only 8.3% did not display restricted diffusion. Additionally, 80.8% exhibited mild perilesional edema, while 76.9% had a mild mass effect. T2W hypointense rims and the central vein sign were observed in approximately half of the patients (45% and 56.5%, respectively). Besides, brain MRI of ten patients revealed the presence of co-existing demyelinating lesions in various brain regions. Representative brain MRI from six patients within the study cohort is illustrated in Fig. 4. Representative brain MRI demonstrating the central vein sign (CVS) and the T2-weighted hypointense rim, is revealed in Fig. 5.

Among those having brain CT performed, hypodensity lesions were found in all cases. MR perfusion studies revealed hyperperfusion in the affected areas in 5 of 7 patients. MR spectroscopy findings are documented in Table S3 of the Supplementary material.

### Pathological data

**Figure 6:**
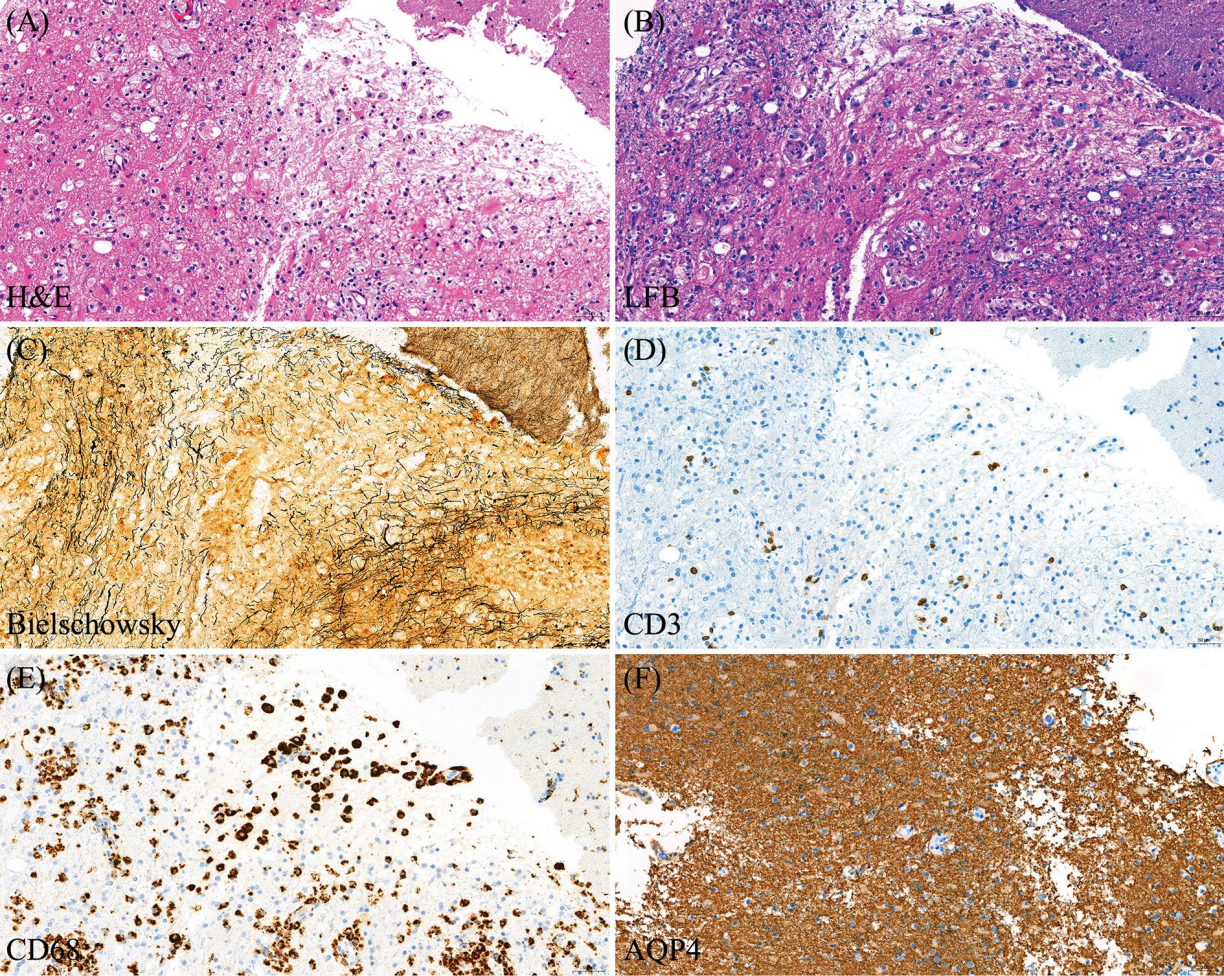
Pathological findings from Patient 4 diagnosed with a single attack of tumefactive demyelinating lesion. (**A**) H&E shows reactive gliosis. (**B**) Luxol fast blue (LFB) highlights small demyelinating areas with some macrophages containing myelin fragments. (**C**) Bielschowsky stain highlights relatively preserved axonal structure. (**D**, **E**) Immunohistochemical study of CD3 and CD68 illustrates that the majority of inflammatory cells are CD68 + T-cells, while a smaller number of CD3 + T-cells are present in the perivascular area and scattered in the tissue. (**F**) Staining for aquaporin-4 (AQP4) is positive.

Brain biopsy was performed on 12 patients (46.2%), with a median interval from symptom onset to biopsy of 58.5 days (range 8–286). The main pathological findings included the combination of reactive astrocytes and perivascular infiltration of lymphocytes (mainly CD3 + T-cells) and myelin-laden macrophages intermingling with the relative preservation of axons within the sharply demarcated lesion. Detailed pathological reports for each case can be found in Table S3 of the Supplementary material. Pathological findings from patient 4 are presented in Fig. 6.

### Other ancillary investigations

Spinal MRI was evaluated in 21 patients, revealing abnormalities in 4 (19%), namely single or multifocal short-segment T2 hyperintense lesions with or without contrast enhancement (Table S3, Supplementary material). Lumbar puncture was performed in all patients, providing complete CSF data in 25 patients. The median CSF white cell count was 2 cells/mm^3^ (range 0–150), and the median CSF protein concentration was 33 mg/dL (range 15–427). Additionally, the median CSF glucose concentration was 63 mg/dL (range 44–110), with a median CSF/serum glucose ratio of 0.6 (based on available data from 16 patients). CSF cytology showed no atypical cell among 24 patients.

Regarding CSF OCBs, 17 out of 21 patients (81%) exhibited type 1 OCBs (absence of bands in both serum and CSF). Type 2 OCBs (detected only in CSF) were observed in three patients (1.4%), while only one patient showed matched bands in both serum and CSF (type 3). Serum AQP4-IgG testing was positive in 4 of 24 patients (16.7%). MOG-IgG testing was conducted in serum (13 cases) and CSF (3 cases), with all results returning negative.

Ophthalmic assessments were done in 22 patients. Eight patients (36.4%) exhibited decreased visual acuity (worse than 6/6), and 3 cases (13.6%) displayed optic disc pallor on fundoscopy. Visual evoked potential was performed in 7 patients, with 2 cases (28.6%) demonstrating delayed P100 latency consistent with demyelination. In addition, optical computed tomography was evaluated in 5 patients, with 2 of them displaying bilateral thinning of macular ganglion cell layer.

### Treatment and prognosis

In terms of acute management (Table S3, Supplementary material), intravenous corticosteroids were administered to 20 patients (76.9%) for a median duration of 5 days (range 3–9). Therapeutic plasma exchange was performed in 9 patients (34.6%), either sequentially or concurrently with intravenous corticosteroid treatment, with a median exchange cycle number of 5 (range 4–7). Intravenous immunoglobulin was administered to only 1 patient. Two patients required decompressive craniectomy, while 5 cases did not receive any acute treatment.

For maintenance therapy, various medications were administered (rituximab in 6 cases, azathioprine in 4 cases, interferon-β1a in 1 case, and teriflunomide in 1 case), while about half of the patients (14 cases, 53.8%) did not receive any long-term medication.

Follow-up MRI data were available for 23 patients, with a median interval between TDL diagnosis and the last follow-up MRI of 3 months (range 0.5–47). All cases showed some degree of lesion resolution.

The median follow-up time was 48 months (range 6–300). The median EDSS score decreased from 4.3 (range 0.0–9.5) at the time of diagnosis to 3.0 (range 0.0–10.0) at the last follow-up visit. Nearly a quarter of patients (23.1%) experienced subsequent demyelinating attacks, with a median time to the next attack of 5 months (range 2–33). More than half (57.7%) had a monophasic course, while the remaining exhibited a relapsing–remitting course. The median number of TDLs attacks and that of any demyelinating attacks were 1 (range 1–3), and 1 (range 1–8), respectively. Unfortunately, one patient (Patient 9 in Table S4 in the Supplementary materials) passed away shortly after hospitalization.

Final diagnoses are summarized in Table 4, only 8 patients (30.8%) met the diagnostic criteria of specific CNS-IDD^28–30^. (4 with relapsing–remitting MS, and 4 with NMOSD with AQP4-IgG), while about two-thirds (69.2%) remained idiopathic, even though some of them developed additional subsequent demyelinating attacks or simultaneous demyelinating syndrome at the other site.

**Table 4:** Clinical course, outcomes, and final diagnosis of 26 patients in the cohort.

Characteristics	N = 26
Total follow-up period^¶^, months	48 (6–300)
Occurrence of subsequent CNS-IDD attacks, n (%)	6 (23.1)
Types of clinical course, n (%)	
Monophasic	15 (57.7)
Relapsing–remitting	11 (42.3)
Total number of TDL attacks^¶^	1 (1–3)
Total number of any CNS-IDD clinical attacks^¶^	1 (1–8)
EDSS at diagnosis^¶^	4.3 (0.0–9.5)
EDSS at the last follow-up visit^¶^	3.0 (0.0–10.0)
Final diagnosis, n (%)	
Single attack TDL	9 (34.6)
Single attack TDL with other CNS-IDD attacks	6 (23.1)
Single attack TDL with other simultaneous CNS-IDD	2 (7.7)
Recurrent attack TDL	1 (3.8)
Relapsing–remitting multiple sclerosis	4 (15.4)
Neuromyelitis optica spectrum disorder with AQP4-IgG	4 (15.4)

AQP4-IgG, aquaporin-4 antibody; CNS-IDD, central nervous system inflammatory demyelinating disease; EDSS, Expanded disability Status Scale; TDL, tumefactive demyelinating lesion.

^¶^Quantitative variables are displayed as the median (range).

## Discussion

TDL, a rare subset of CNS-IDD, pose a diagnostic challenge due to their similarity to true mass lesions, often resulting in delayed investigations and treatments. Despite their clinical significance, there is a lack of data regarding their pathophysiology, standard treatment protocols, prognostic factors, and optimal follow-up strategies. A comprehensive understanding of TDL is essential for guiding therapeutic development. Moreover, the prevalence of specific diseases within the spectrum of CNS-IDD varies geographically, possibly influenced by genetic and environmental factors, necessitating broader studies across diverse regions and ethnic groups. This retrospective cohort study in Thailand found that TDL accounted for 2.4% of cases in two tertiary hospitals. TDL manifested as MS, NMOSD with AQP4-IgG, and isolated demyelinating events, with no cases showing MOG-IgG seropositivity. TDL remarkably served as the initial presentation of demyelinating disease in 77% of cases. Over half of the patients (57.7%) exhibited monophasic courses, both with and without long-term treatments.

Studies have reported the prevalence of TDL within the context of various diseases that exhibit TDL manifestations, revealing incidence rates such as 1–2 per 1000 cases of MS^31–35^, 5 per 100 cases of NMOSD with AQP4-IgG^20^, and 22 per 100 cases of MOGAD^20^. In contrast, our present study encompassed all patients with TDL, characterized by their minimum 2 cm diameter, mass-like features, and underlying inflammatory demyelination, across their disease course. This comprehensive approach yielded a TDL prevalence of 2.4 per 100 individuals within the CNS-IDD spectrum. The median age at TDL onset in our study (34.5 years, range 17–75) closely aligned with previous findings, where TDL onset typically occurred in late adolescence to middle adulthood (20 s-30 s)^19,36–38^. Notably, our study revealed an intriguing observation, with 3 out of 4 patients presenting with TDL during their initial CNS-IDD attack, a higher proportion than reported in previous studies^19,24,38,39^.

In terms of clinical presentation, this study strengthened the observation that motor and sensory symptoms were the predominant features in TDL. Cognitive symptoms, indicative of cortical involvement, were observed in a quarter of cases. Headache, often linked to intracranial hypertension, was relatively rare, occurring in only 16% of patients, which could be attributed to the generally modest size of TDL in our cohort (with 69.2% having a diameter of < 5 cm). Many patients exhibited a polysymptomatic presentation, attributable to the lesions themselves and perilesional edema causing neurological dysfunction in affected areas. Nevertheless, in the majority of patients, symptoms could still be localized to a specific anatomical region (monofocal), primarily due to the solitary nature of most TDL in this study. These clinical findings aligned with previous research^40,41^. Regarding the median duration from initial hospital visit to definitive diagnosis, which averaged 1 month with a maximum of 9 months, this underscored the issue of delayed diagnosis among TDL patients, potentially impacting treatment and outcomes.

As expected, the most prevalent locations for TDL in this study were the fronto-parietal regions, followed by the frontal region. This aligned with the fact that the frontal and parietal lobes collectively constitute over 40% of the entire brain mass^42^. Basal ganglia and thalamus were involved in 19.2% of cases, a rate higher than reported in some prior studies^20,24,36,43^. Notably, a large retrospective cohort in China similarly found deep grey nuclei involvement in 35% of cases^25^. In terms of gadolinium-enhancing patterns, the open ring pattern, known for its high specificity in diagnosing atypical demyelinating lesions^44,45^, was prevalent in half of the patients, exceeding previous reports^19,46^. This high frequency of open ring patterns may be attributed to the subacute clinical course observed in the cohort. Studies by Song et al. have highlighted dynamic enhancement patterns in TDL, with ring enhancement more common during the subacute phase^47^. This current study also noted restricted diffusion in 91.7% of the patients, primarily in the peripheral areas, consistent with prior research^36,41^. Co-existing demyelinating lesions were identified from brain MRI in 38.5% of patients, and from spinal MRI in 19% of patients, although these could not fulfill the diagnostic criteria of RRMS due to TDL not being counted as one region in the 'dissemination in space' criteria of the 2017 McDonald criteria^28^. Nevertheless, this finding underscored the presence of demyelinating lesions distributed across multiple brain regions. While multiple asymptomatic demyelinating lesions have been associated with an increased risk of conversion to MS^21^, this study, limited by its small cohort size and relatively short follow-up period, cannot evaluate this as a prognostic factor.

In less than half of the study patients, a pathological diagnosis was deemed necessary, as conclusive diagnoses could generally be established through clinical evaluation, radiological findings^22^, and treatment responses. Brain biopsy was reserved for cases with equivocal investigation results, contributing to the median delay in the interval from symptom onset to biopsy in this study. Among other ancillary investigations, the detection of OCBs was observed in only 19% of the patients, a finding consistent with studies in China^25^ and Korea^48^ but lower than those conducted in the USA^20,49^ and Europe^36,39,43,50^. This disparity in OCBs detection can be attributed to the higher prevalence of MS patients in the USA and European studies. It is worth noting that only half of the cohort underwent testing for serum MOG-IgG, with 11.5% undergoing CSF MOG-IgG testing, as these tests had only recently become available in routine clinical practice in Thailand within the last three years. The recent diagnostic criteria for MOGAD highlight the utility of isolated CSF MOG-IgG positivity in specific situations to support the diagnosis of MOGAD in MOG-IgG seronegative patients exhibiting compatible clinical and MRI features^29^. Additionally, Cacciaguerra et al. have reported a higher frequency of TDL among MOGAD patients^20^, underscoring the importance of appropriate MOG-IgG testing in both serum and CSF for diagnosing MOGAD in TDL patients.

The majority of patients in our study cohort underwent acute management following established TDL management algorithms, in line with prior recommendations^21,51^. This typically involved intravenous corticosteroid treatment for 3–7 days, followed by therapeutic plasma exchange for those who did not respond to corticosteroids. However, five patients did not receive acute treatment. One patient did not receive acute treatment due to a mild neurological deficit and was lost to follow-up. Another patient was initially misdiagnosed with a low-grade glioma but remained stable without further attacks. The reasons for the remaining three cases not receiving acute treatment were unspecified. In terms of maintenance therapy, half of the cohort received specific treatments. This included four patients diagnosed with RRMS, three with NMOSD with AQP4-IgG, and one with a single TDL and a history of bilateral optic neuritis. Additionally, four patients diagnosed with single attack TDL chose to undergo long-term medication based on discussions with their physicians.

The prognosis for TDL patients in this study was favorable. Over a 48-month median follow-up, four patients developed RRMS, and three had NMOSD with AQP4-IgG. Among the remaining eighteen cases, only two patients experienced subsequent CNS-IDD attacks, a lower rate compared to previous studies^43^. The median time between TDL and a subsequent attack was 5 months, emphasizing the need for early post-TDL reevaluation. Sixty-five percent showed improved EDSS scores, while 26.9% maintained stable scores. It is worth emphasizing that EDSS scores primarily reflect physical disability and have only a weak correlation with cognitive function^52^. Hence, future research should prioritize long-term cognitive assessments in TDL patients.

This study has some limitations. Firstly, its retrospective nature led to some missing data. Secondly, the relatively small patient cohort posed challenges in exploring prognostic factors or conducting inferential analyses. Nevertheless, our primary aim was to comprehensively describe the characteristics of TDL patients. Thirdly, the follow-up duration was relatively short, underscoring the potential need for long-term observation to solidify patient diagnoses. Lastly, our patient group was heterogeneous, encompassing all individuals with at least one TDL attack. Despite these limitations, this research establishes a fundamental comprehension of TDL within the context of Thailand and serves as a foundation for future research, ultimately contributing to improved patient care and outcomes.

## Conclusion

TDL remain a rare occurrence among Thai CNS-IDD patients, and this study revealed that the clinical, radiological, and laboratory features of Thai TDL patients are consistent with previous cohorts. We identified four cases of RRMS and four cases of NMOSD with AQP4-IgG among the study patients. The median two-year follow-up showed a favorable prognosis, with only two out of eighteen isolated TDL patients experiencing subsequent CNS-IDD attacks. To deepen our understanding of prognostic factors, larger prospective cohorts are imperative.

### Supplementary Information

Supplementary Information.

## Data Availability

Data supporting the findings are available upon request from the corresponding authors.
