# Author Correction: Tumefactive demyelinating lesions: a retrospective cohort study in Thailand

**DOI:** 10.1038/s41598-024-56016-2

**Published:** 2024-03-04

**Authors:** Tatchaporn Ongphichetmetha, Saharat Aungsumart, Sasitorn Siritho, Metha Apiwattanakul, Jantima Tanboon, Natthapon Rattanathamsakul, Naraporn Prayoonwiwat, Jiraporn Jitprapaikulsan

**Affiliations:** 1 Division of Neurology, Department of Medicine, Faculty of Medicine Siriraj Hospital, Mahidol University, 2 Wanglang Rd, Siriraj, Bangkok‑noi, Bangkok, 10700 Thailand; 2 Siriraj Neuroimmunology Center, Faculty of Medicine Siriraj Hospital, Mahidol University, Bangkok, 10700 Thailand; 3 Division of Clinical Epidemiology, Department of Research and Development, Faculty of Medicine Siriraj Hospital, Mahidol University, Bangkok, 10700 Thailand; 4 Neuroimmunology Unit, Department of Neurology, Neurological Institute of Thailand, Bangkok, 10400 Thailand; 5 Bumrungrad International Hospital, Bangkok, 10110 Thailand; 6 Department of Pathology, Faculty of Medicine Siriraj Hospital, Mahidol University, Bangkok, 10700 Thailand

Correction to: *Scientific Reports* 10.1038/s41598-024-52048-w, published online 16 January 2024

The original version of this Article contained an error, where the Certificate of Approval (COA) number provided in the published version is incorrect.

As a result, in the section, “Methods”,

“The study was approved by Siriraj Institutional Review Board (EC 299/2566) and the Institutional Review Board of the Neurological Institute of Thailand (approval number 60026) and all methods were performed in accordance with the guidelines and regulations.”

now reads:

“The study was approved by Siriraj Institutional Review Board (COA no. Si 391/2023) and the Institutional Review Board of the Neurological Institute of Thailand (approval number 66037) and all methods were performed in accordance with the guidelines and regulations.”

The original Article has been corrected.

