# Supplementary material for: Tumefactive demyelinating lesions: a retrospective cohort study in Thailand
Source: Sci Rep. 2024 Jan 16;14:1426. doi: 10.1038/s41598-024-52048-w (PMC10791607; doi:10.1038/s41598-024-52048-w)
Supplement: Supplementary file 1 — Supplementary Information. [file 41598_2024_52048_MOESM1_ESM.docx]

**Supplementary table S1.** Clinical data of the study cohort

| **Case No.** | **Baseline characteristics** | | | | **TDL: Clinical data** | | | | | |
| --- | --- | --- | --- | --- | --- | --- | --- | --- | --- | --- |
|  | **Sex** | **Previous CNS-IDD diagnosis** | **Co-morbidities** | **Previous neurological status** | **Age at onset, years** | **Clinical presentation** | **Domains of clinical presentation** | **Time from symptom onset to first hospital visit, days** | **Time from first hospital visit to diagnosis, days** | **Risk factors of CNS-IDD** |
| 1 | Female | None | None | Normal | 36 | Subacute progressive left hemihypesthesia with intermittent left arm tonic activity with right facial weakness with motor aphasia (3 months) | Sensory, motor symptom and cognitive problem (polyfocal) | 5 | 150 | None |
| 2 | Male | None | None | Normal | 35 | Subacute progressive headache (1 month) followed by tonic-clonic seizures of the right-sided mouth progressing to generalized tonic-clonic seizures | Headache and seizures (monofocal) | 30 | 7 | History of fever (1 month prior) |
| 3 | Female | None | None | Normal | 33 | Subacute progressive left leg monoparesis with decreased mental status (6 days) | Motor symptoms, and consciousness problem (polyfocal) | 2 | 11 | History of viral gastroenteritis  (2 weeks prior) |
| 4 | Male | None | None | Normal | 32 | Tonic seizures of the left arm with decreased mental status followed by left leg monoparesis and hypoesthesia (2 weeks) | Motor and sensory symptoms, seizures, and consciousness problem (monofocal) | 0 | 293 | None |
| 5 | Male | None | None | Normal | 20 | Subacute progressive left hemiparesis and hemihypesthesia (2 months) | Motor and sensory symptoms (monofocal) | 58 | 4 | None |
| 6 | Male | None | None | Normal | 36 | Subacute progressive left hemiparesis and hemihypesthesia (1 month) | Motor and sensory symptoms (monofocal) | 4 | 30 | None |
| 7 | Female | None | None | Normal | 29 | Subacute progressive left hemiparesis and hemihypesthesia (2 weeks) | Motor and sensory symptoms (monofocal) | 10 | 43 | None |
| 8 | Male | None | None | Normal | 53 | Subacute progressive left hemiparesis with altered mental status (2 months) | Motor symptom and consciousness problem (monofocal) | 60 | 39 | None |
| 9 | Female | None | Hypertension | Normal | 73 | Subacute progressive paraphasia followed by altered mental status (4 months) | Cognitive and consciousness problem (monofocal) | 30 | 90 | None |
| 10 | Female | Single attack ON (3 years prior, no IS) | Allergic rhinitis | Left eye visual loss (VA 6/30, RAPD positive) | 23 | Subacute progressive left hemiparesis (1 month) | Motor symptom (monofocal) | 30 | 42 | None |
| 11 | Female | None | None | Normal | 25 | Subacute progressive right hemiparesis with motor aphasia with decreased mental status (1.5 months) | Motor symptom, cognitive problem, and consciousness problem (monofocal) | 1 | 76 | None |
| 12 | Female | Single attack bilateral optic neuritis (1 month prior) | Diabetes mellitus, hypertension, dyslipidemia | None | 75 | Asymptomatic (TDL were found during follow-up orbital MRI) | - | - | - | None |
| 13 | Female | Single attack longitudinally extensive transverse myelitis (10 years prior) | None | Normal | 69 | Chronic progressive right hemiparesis with left hemineglect with dressing apraxia (6 months) | Motor symptom and cognitive problem (polyfocal) | 180 | 60 | Recently received the third dose of BNT162b2 COVID-19 vaccination (1 month prior) |
| 14 | Female | None | None | Normal | 26 | Subacute progressive right hemiparesis with motor aphasia with headache (1 month) followed by generalized tonic-clonic seizures | Motor symptom, cognitive problem, headache, and seizures (monofocal) | 30 | 49 | None |
| 15 | Female | None | Hypertension | Normal | 61 | Subacute progressive right hemihypesthesia  (1 week) | Sensory symptoms (monofocal) | 7 | 2 | None |
| 16 | Male | None | None | Normal | 25 | Subacute progressive left hemiparesis with hemihypesthesia with monocular visual loss of the left eye with diffuse headache (2 months) | Motor, sensory, visual symptoms, and headache (polyfocal) | 7 | 90 | None |
| 17 | Male | None | First diagnosis of HIV infection | Normal | 22 | Subacute progressive left-sided visual field defect with psychomotor retardation (1 month) | Visual symptoms and consciousness problem (polyfocal) | 15 | 10 | Recent COVID-19 infection  (1 month prior) |
| 18 | Female | None | None | Normal | 59 | Subacute progressive right hemiparesis with dysarthria (2 months) | Motor symptom (monofocal) | 60 | 12 | None |
| 19 | Female | Single attack transverse myelitis (19 years prior, no IS) | None | Normal | 34 | Subacute progressive left hemiparesis (2 week) | Motor symptom (monofocal) | 7 | 133 | None |
| 20 | Male | None | None | Normal | 37 | Subacute progressive left hemiparesis (3 months) | Motor symptom (monofocal) | 58 | 6 | None |
| 21 | Female | Single attack transverse myelitis (9 months prior) | Thalassemia trait | Normal | 37 | Subacute progressive left hand dystonia with left hemiparesis and hemihypesthesia with urinary incontinence (1 month) | Motor and sensory symptoms, movement disorder, and bladder dysfunction (polyfocal) | 30 | 45 | None |
| 22 | Female | None | None | Normal | 17 | Subacute progressive right hemiparesis with hemihypesthesia  (1 months) | Motor and sensory symptoms (monofocal) | 10 | 14 | None |
| 23 | Female | None | Breast cancer | Normal | 43 | Subacute progressive right hand monoparesis with motor aphasia with monocular visual loss of the right eye (1 month) with intermittent right arm tonic-clonic activity | Motor and visual symptom, cognitive problem, and seizures (polyfocal) | 30 | 14 | None |
| 24 | Female | Neuromyelitis optica spectrum disorder with AQP4-IgG (Single attack brainstem syndrome with 3 recurrent optic neuritis) | None | Right eye visual loss (VA hand motion) | 32 | Subacute progressive left-sided visual field defect and left foot numbness (2 weeks) followed by generalized tonic-clonic seizures | Sensory and visual symptoms, and seizures  (monofocal) | 6 | 91 | None |
| 25 | Female | None | None | Normal | 43 | Subacute progressive intractable hiccup with paraphasia with fever followed by quadriparesis (1 week) | Brainstem (area postrema syndrome) and cognitive problem and motor symptom (polyfocal) | 7 | 7 | None |
| 26 | Female | None | None | Normal | 32 | Subacute progressive quadriparesis with binocular horizontal diplopia with right face hemihypesthesia (3 months) | Motor and brainstem symptoms (monofocal) | 4 | 26 | History of fever with headache (1 month prior) |

Abbreviations: AQP4-IgG, aquaporin-4 antibody; CNS-IDD, inflammatory demyelinating diseases of the central nervous system; COVID-19, coronavirus disease 2019; HIV, human immunodeficiency virus; IS, immunosuppressive agents; MRI, magnetic resonance imaging; ON, optic neuritis; RAPD, relative afferent papillary defect; TDL, tumefactive demyelinating lesions; VA, visual acuity.

**Supplementary table S2.** MRI data of Thai tumefactive demyelinating lesion cohort

| **Patient No.** | **TDL: Lesion characteristics on Brain MRI** | | | | | | | | | | | |
| --- | --- | --- | --- | --- | --- | --- | --- | --- | --- | --- | --- | --- |
|  | **Time from onset to MRI, days** | **Lesion number** | **Lesion location** | **Lesion size, cm^3^** | **Pattern of gadolinium enhancement** | **Restricted diffusion** | **Perilesional edema** | **Mass effect** | **Central vein sign** | **T2W hypointense rim** | **Other co-existing demyelinating lesions** | **Other MRI characteristics** |
| 1 | 150 | 2 | Left frontal extending to and right parietal | 2.7x3.6x2.7  and 1.8x2.2x2.2 | Heterogenous | Peripheral restricted diffusion | Mild | Mild | Present | Present | None | Solid-cystic lesion at left frontal region |
| 2 | 35 | 1 | Left fronto-temporal | 4.4x3.6x4.5 | Open ring | Peripheral restricted diffusion | Moderate | Moderate | None | Present | None | - Balo-like lesion  - Blooming artifact on SWI |
| 3 | 6 | > 10 | Bilateral cerebral hemisphere, thalamus, and left cerebral peduncle | Each lesion diameter of up to 2 cm | Open ring | Peripheral restricted diffusion | Mild | Mild | None | Present in some lesions | None | - Same stage of all lesions  - Involvement of splenium of corpus callosum |
| 4 | 8 | 1 | Right fronto-parietal | 2.4x2.2x2.9 | Patchy | Peripheral  Restricted diffusion | Mild | Mild | None | None | None | None |
| 5 | 62 | 1 | Right fronto-parietal | 3.6x3.1x3.9 | Homogenous | Peripheral restricted diffusion | Mild | Mild | Present | Present | None | None |
| 6 | 30 | 1 | Right fronto-parietal | 4.5x4.9x4.2 | Heterogenous | Peripheral restricted diffusion | Mild | Mild | Present | Present | None | None |
| 7 | 10 | 1 | Right fronto-parietal extending to right basal ganglia | 8.0x5.0x7.0 | Nodular | No restricted diffusion | Moderate | Moderate | ND | ND | None | None |
| 8 | 72 | 1 | Right fronto-parietal | 6.0x5.0x5.0 | Patchy | Peripheral restricted diffusion | Moderate | Mild | Present | None | None | None |
| 9 | 160 | 4 | Left frontal, left temporo-occipital, right frontal, right parieto-occipital, | 6.4x6.2x4.8  and  4.0x1.9x2.4 and 3.4x2.1x3.9 and 3.5x2.2x2.5 | Heterogenous | Peripheral restricted diffusion | Mild | Mild | None | Present | None | Involvement of anterior genu of left corpus callosum |
| 10 | 30 | 1 | Right parieto-temporo-occipital | 3.0x3.1x2.9 | Homogenous | Central restricted diffusion (in the gadolinium-enhancing region of the lesion) | Moderate | Moderate | ND | None | None | Involvement of splenium of corpus callosum |
| 11 | 54 | 1 | Left basal ganglia and thalamus | 5.4x5.3x5.1 | Open ring | Peripheral restricted diffusion | Moderate | Moderate | ND | None | None | None |
| 12 | - | 2 | Left frontal and right fronto-parietal | 2.2x2.2x1.8 and 3.3x3.8x3.7 | Open ring | Peripheral restricted diffusion | Mild | Mild | Present | Present | Bilateral optic nerve (with gadolinium-enhancement) (same), right thalamus (with gadolinium-enhancement) (new lesion) | None |
| 13 | 180 | 2 | Bilateral temporo-occipital | 3.1x3.3x1.4 and 3.2x1.8x2.2 | Open ring | Peripheral restricted diffusion | Mild | Mild | Present | Present | None | Involvement of splenium of corpus callosum |
| 14 | 56 | 3 | Bilateral fronto-parietal, left frontal | 5.7x4.6x5.9 and 4.8x3.5x4.4 and 4.2x2.6x3.8 | Open ring | Peripheral restricted diffusion | Mild | Mild | Present | Present | None | None |
| 15 | 9 | 1 | Left cerebellar peduncle and left-sided pons | 2.4x2.4x2.3 | Open ring | Peripheral restricted diffusion | Mild | Mild | None | Present | None | None |
| 16 | 60 | 2 | Right frontal extending to right basal ganglia and left frontal | 4.2x6x3.1 and 1.8x3x2.1 | Open ring | ND | Mild | Moderate | Present | Present | Left optic nerve (with gadolinium-enhancement) | Balo-like lesion |
| 17 | 15 | 4 | Bilateral frontal, left fronto-parietal, right parieto-occipital | 3.8x4.7x4.9and 1.7x1.7x2.0 and 3.9x3.7x4.5 and 4.5x3.2x4.0 | Open ring | Peripheral restricted diffusion | Mild | Mild | None | None | Bilateral optic nerve (with gadolinium-enhancement) | Involvement of anterior genu of right corpus callosum |
| 18 | 70 | 1 | Left fronto-parietal | 3.5x3.0.4.0 | Patchy | Peripheral restricted diffusion | Mild | Mild | ND | ND | None | None |
| 19 | 7 | 1 | Right fronto-parietal | 4x5x4 | Open ring | ND | Mild | Mild | ND | None | Periventricular, juxtacortical, midbrain | None |
| 20 | 58 | 1 | Right frontal | 4.6x4.7x3.1 | Heterogenous | Peripheral restricted diffusion | Moderate | Mild | Present | None | Periventricular (1 lesion with gadolinium-enhancement) | Balo-like lesion |
| 21 | 30 | 2 | Left fronto-parietal | 2.6x2.1x2.2 and 2.0x1.1x0.8 | Closed ring | Peripheral restricted diffusion | Mild | Mild | Present | Present | Right ventral medulla (with gadolinium-enhancement), left cerebral peduncle, right ventral pons, multiple Periventricular | None |
| 22 | 24 | 1 | Left-sided thalamus | 2.1x1.9x1.7 | Open ring | Peripheral restricted diffusion | Mild | Mild | None | None | Periventricular | None |
| 23 | 30 | 2 | Left fronto-parietal and right temporal | 4.9x4.9x6.9  and 1.7x2x0.8 | Patchy | Peripheral restricted diffusion | Mild | Moderate | None | Present | Right optic nerve | None |
| 24 | 20 | 1 | Right thalamus | 2.1x1.5x2.1 | Heterogenous | No restricted diffusion | Mild | Mild | None | None | Temporal horn of right lateral ventricle (with gadolinium-enhancement) | None |
| 25 | 7 | 1 | Right-sided paramedian ventral pons and cerebral peduncle | 2.8x1.7x2.2 | Open ring | Peripheral restricted diffusion | Mild | Mild | None | None | Dorsal medulla, paramedian thalamus, left sided hypothalamus, left fornix, subcortical and deep white matter of both frontoparietal lobe | None |
| 26 | 30 | 1 | Pons and bilateral middle cerebellar peduncle and bilateral cerebral peduncle and left posterior limb of the internal capsule | 2.8x2.8x2.6 | Open ring | Peripheral restricted diffusion | Mild | Mild | None | None | None | None |

Abbreviations: MRI, magnetic resonance imaging; ND, no data; T2W, T2-weighted imaging; TDL, tumefactive demyelinating lesions; SWI, susceptibility-weighted imaging.

**Supplementary table S3.** Data of other investigations and management of Thai tumefactive demyelinating lesion cohort

| **Case No.** | **TDL: Lesion characteristic in other imaging modalities** | | | **TDL: Pathology** | | **TDL: Other investigations** | | | | | | |
| --- | --- | --- | --- | --- | --- | --- | --- | --- | --- | --- | --- | --- |
|  | **CT brain** | **MR Perfusion** | **MR Spectroscopy** | **Time from onset to biopsy, days** | **Findings** | **Spinal cord MRI** | **Cerebrospinal fluid profile (CSF)** | | | | | **OCBs** |
|  |  |  |  |  |  |  | **OP, cmH_2_O** | **WBC and differential, cells/mm^3^** | **Protein, g/dL** | **Glucose (CSF/blood), g/dL** | **CSF cytology** |  |
| 1 | Hypodensity | ND | Decreased NAA/Cr ratio with increased Cho/Cr and Cho/NAA ratio, mild increased lactate peak | 165 | - Sharply circumscribed lesion consisting of a mixture of reactive astrocytes and perivascular lymphocyte and macrophage infiltration  - Myelin material in macrophages with relatively preserved axonal process | Normal | 17.5 | 1 | 31 | 59/95 | No atypical cell | Type 1 |
| 2 | Hypodensity | Hypoperfusion | Increased Cho and lipid/lactate peak, and decreased NAA | 37 | Perivascular infiltration of CD3+ lymphoid cells and myelin-containing histiocytes with relatively preserved axon | Normal | 13 | 5 (lymphocyte 99%, monocyte 1%) | 26 | 55/106 | No atypical cell | Type 1 |
| 3 | Hypodensity | ND | ND | ND | ND | Normal | 19.5 | 0 | 427 | 66/91 | No atypical cell | Type 1 |
| 4 | ND | Hyperperfusion | High Cho/Cr ratio and low NAA | 286 | Perivascular infiltration of myelin-containing macrophages with smaller number of T-cells, reactive astrocytes, small demyelinating areas with relatively preserved axon | Normal | 18 | 0 | 30 | 62/108 | No atypical cell | Type 1 |
| 5 | ND | ND | ND | ND | ND | Normal | ND | 3 (monocyte 100%) | 15 | 64/ND | No atypical cell | ND |
| 6 | ND | ND | High Cho, low NAA, Cho/NAA ratio 5.27, prominent lactate peak | ND | ND | ND | 14 | 0 | 40 | 87/ND | No atypical cell | ND |
| 7 | Hypodensity | ND | ND | ND | ND | Normal | ND | 0 | 19 | 69/ND | No atypical cell | ND |
| 8 | ND | ND | High Cho | ND | ND | Normal | ND | 1 | 29 | 97/192 | No atypical cell | Type 2 |
| 9 | Hypodensity | ND | ND | 40 | Presence of active inflammation (predominantly CD3+ small lymphoid cells and histiocytes) and reactive gliosis | ND | ND | 2 (neutrophil 50%, lymphocyte 50%) | 47 | 87/ND | No atypical cell | ND |
| 10 | Minimal enhancing ill-defined hypodensity mass | Hyperperfusion | Borderline Cho/Cr ratio and increased lactate peak | 45 | Presence of reactive astrocytes with perivascular lymphocyte cuffing and macrophage | ND | 16 | 1 | 33 | 63/102 | No atypical cell | Type 1 |
| 11 | Hypodensity | ND | No definite rising of Cho/Cr ratio or decreased NAA peak, rising of lipid/lactate complex | 55 | Fragments of white matter tissue with pale myelin staining, dense macrophages infiltration, and some reactive astrocytes with rather preserved axon | Normal | ND | ND | ND | ND/ND | No atypical cell | Type 1 |
| 12 | ND | Hyperperfusion | ND | ND | ND | Normal | ND | 2 | 61 | 71/142 | No atypical cell | Type 1 |
| 13 | ND | ND | ND | 210 | Proliferation of foamy histiocytes, reactive gliosis, and mild perivascular lymphocyte cuffing | Normal | ND | 150 (monocyte 12%, neutrophil 88%) | 251 | 110/125 | No atypical cell | Type 1 |
| 14 | Hypodensity | ND | ND | ND | ND | Normal | 30 | 0 | 23 | 56/ND | No atypical cell | Type 1 |
| 15 | Hypodensity | Hypoperfusion | Slightly increased Cho and Lipids/Lactate peak | ND | ND | Normal | 17 | 2 | 106.3 | 86.9/121 | No atypical cell | Type 1 |
| 16 | Rim enhancing hypodensity lesion | ND | Decreased NAA peak, high Cho peak, increased lipid/lactate peak | 8 | Reactive gliosis with lymphocyte and foamy macrophage infiltrate | Normal | 26 | 3 | 60 | 45/117 | No atypical cell | Type 1 |
| 17 | ND | ND | ND | ND | ND | ND | ND | 0 | 76 | 63/ND | ND | Type 2 |
| 18 | ND | ND | High Cho metabolite | ND | ND | Normal | ND | 0 | 21 | 57/99 | No atypical cell | ND |
| 19 | ND | ND | ND | ND | ND | Normal | 9 | 1 | 94 | 73/105 | No atypical cell | Type 1 |
| 20 | ND | Hyperperfusion | High Cho/NAA ratio | 62 | Sharp demarcation of demyelination with preserved axons with presence of perivascular lymphocyte infiltrate | Multiple levels of abnormal hypersignal T2W lesions along cervical and thoracic spinal cord without abnormal enhancement | 14 | 0 | 22 | 99.1/ND | No atypical cell | Type 1 |
| 21 | Hypodensity mass with rim-enhancement | ND | ND | 77 | Proliferation of lymphoid cells and foamy histiocytes with relatively preserved axon | Multiple levels of abnormal hypersignal T2W lesions along cervical and thoracic spinal cord with abnormal enhancement in some lesions | ND | 12 (mononuclear 100%) | 33 | 58/88 | No atypical cell | Type 2 |
| 22 | ND | ND | ND | ND | ND | Normal | 19 | 2 | 18 | 51/90 | ND | Type 1 |
| 23 | Hypodensity | ND | ND | 32 | Ill-defined necrotic areas composed of many neutrophils and foamy macrophages infiltrate with hypervascularity and increase fibroblastic activity, and evidence of reactive gliosis change | ND | 6.5 | 4 | 36 | 57/94 | No atypical cell | Type 1 |
| 24 | Hypodensity | Hyperperfusion | High Cho and low NAA peak, peak Cho/Cr ratio about 2.1 and 1.6 | 90 | Numerous macrophages infiltrating the white matter, particularly in the perivascular region, and accompanying small lymphocytes with some reactive astrocytes | Hypersignal T2W lesions at T10 spinal cord without gadolinium-enhancement | 12 | 22 (lymphocyte 100%) | 34 | 44/96 | No atypical cell | Type 3 |
| 25 | Hypodensity | ND | ND | ND | ND | Hypersignal T2W lesions at T1-T3 spinal cord with faint gadolinium-enhancement | ND | 5 (mononuclear 100%) | 29.8 | 77.3/ND | No atypical cell | Type 1 |
| 26 | Hypodensity | ND | ND | ND | ND | Normal | ND | 52 (lymphocyte 77%, eosinophil 10%) | 44 | 57/ND | No atypical cell | Type 1 |

Abbreviations: Cho, choline; cm^3^, cubic centimeter; cmH_2_O, centimeter of water; Cr, creatine; CSF, cerebrospinal fluid; CT, computed tomography; g/dL, grams per deciliter; mm^3^, cubic millimeter; MRI, magnetic resonance imaging; MR spectroscopy, magnetic resonance spectroscopy; NAA, N-Acetylaspartic acid; ND, no data; OCBs, oligoclonal bands; OP, open pressure; T2W, T2-weighted imaging; TDL, tumefactive demyelinating lesions; WBC, white blood cell.

**Supplementary table S3.** Data of other investigations and management of Thai tumefactive demyelinating lesion cohort

| **Case No.** | **TDL: Other investigations** | | | | | | | | | **TDL: Management** | | |
| --- | --- | --- | --- | --- | --- | --- | --- | --- | --- | --- | --- | --- |
|  | **Serum AQP4-IgG** | **Serum MOG-IgG** | **CSF AQP4-IgG** | **CSF MOG-IgG** | **Other systemic autoantibodies** | **Ophthalmic examination** | | | | **Acute management** | **Duration of corticosteroid tapering, months** | **Maintenance therapy** |
|  |  |  |  |  |  | Visual function | Fundoscopy | VEP | OCT |  |  |  |
| 1 | Negative | ND | Negative | ND | None | VA 6/6 both eyes | Normal | Normal | ND | No treatment | Not receive corticosteroid | Interferon β-1a |
| 2 | Negative | ND | Negative | ND | None | VA 6/6 both eyes | Normal | Normal | ND | Dexamethasone 5 mg intravenous every 6 hours (3 days) | No corticosteroid tapering | None |
| 3 | Negative | ND | Negative | ND | None | ND | Normal | ND | ND | - Methylprednisolone 1 g intravenous daily (7 days)  - Therapeutic plasma exchange 1 plasma volume (7 cycles) | 4.5 | Natalizumab (8 months) then teriflunomide |
| 4 | Negative | Negative | ND | ND | None | ND | ND | ND | ND | No treatment | Not receive corticosteroid | None |
| 5 | Negative | ND | ND | ND | None | VA 6/6 both eyes | Normal | ND | ND | Methylprednisolone 1 g intravenous daily (5 days) | No corticosteroid tapering | None |
| 6 | Negative | Negative | ND | ND | None | VA 6/6 both eyes | Normal | ND | ND | Methylprednisolone 1 g intravenous daily (5 days) | No corticosteroid tapering | None |
| 7 | Negative | ND | ND | ND | None | VA 6/6 both eyes | Normal | Normal | ND | Dexamethasone 4 mg intravenous every 8 hours (6 days) | No corticosteroid tapering | None |
| 8 | ND | ND | ND | ND | None | VA 6/6 both eyes | Normal | ND | ND | No treatment | Not receive corticosteroid | None |
| 9 | Negative | ND | ND | ND | None | ND | ND | ND | ND | Dexamethasone 5 mg intravenous every 6 hours (9 days) | No corticosteroid tapering | None |
| 10 | ND | ND | ND | ND | Anti-SSA positive 2+ | Left eye VA 6/30 (same) | ND | ND | ND | Dexamethasone 5 mg intravenous every 8 hours (6 days) | 1 | None |
| 11 | Negative | ND | ND | ND | None | VA 6/6 both eyes | Normal | Delayed P100 latency with moderate decreased amplitude of both eyes | Thinning retina nerve fiber layer of both eyes with thinning retina ganglion cell layer of the right eye | Decompressive craniectomy with ventriculostomy | Not receive corticosteroid | Azathioprine |
| 12 | Negative | Negative | ND | ND | ANA positive 1:100 (fine-speckled, nucleolar pattern) | VA finger count at 1 foot with RAPD positive of the left eye, finger count at 2 feet right eye | Mildly pale optic disc both eyes | ND | ND | - Methylprednisolone 1 g intravenous daily (5 days)  - Therapeutic plasma exchange 1 plasma volume (5 cycles) | 6 | Azathioprine |
| 13 | Negative | Negative | Negative | Negative | None | VA 6/6 both eyes | Normal | ND | ND | - Methylprednisolone 1 g intravenous daily (5 days)  - Therapeutic plasma exchange 1 plasma volume (5 cycles) | 6 | None |
| 14 | Negative | Negative | Negative | Negative | None | VA 6/30 right eye, 6/24 left eye, RAPD negative both eyes | Normal | Delayed P100 latency of both eyes | No thinning of retinal nerve fiber layer or ganglion cell layer | - Methylprednisolone 1 g intravenous daily (5 days)  - Therapeutic plasma exchange 1 plasma volume (7 cycles) | 6 | None |
| 15 | Negative | Negative | ND | ND | None | VA 6/6 both eyes | Normal | ND | ND | Methylprednisolone 1 g intravenous daily (3 days) | 0.5 | None |
| 16 | Negative | ND | Negative | ND | None | VA 6/36 with RAPD positive of the left eye, 6/24 right eye | Normal | ND | Bilateral macular ganglion cell thickness thinning (left eye > right eye) | - Craniectomy with tumor removal  - Methylprednisolone 1 g intravenous daily (5 days) | 17 | Azathioprine |
| 17 | Negative | Negative | Negative | Negative | ANA positive 1:100 (fine-speckled, nucleolar pattern) | VA finger count at 1 foot both eyes with RAPD negative both eyes | Normal | ND | ND | - Methylprednisolone 1 g intravenous daily (6 days)  - Therapeutic plasma exchange 1 plasma volume (4 cycles) | 7 | Azathioprine |
| 18 | Negative | Negative | ND | ND | None | VA 6/6 both eyes | Normal | ND | ND | No treatment | Not receive corticosteroid | None |
| 19 | Negative | ND | Negative | ND | None | VA 6/7.5 left eye, 6/6 right eye, RAPD negative both eyes | Normal | ND | ND | No treatment | Not received corticosteroid | None |
| 20 | Negative | Negative | ND | ND | ND | VA 6/6 both eyes | Normal | ND | ND | - Methylprednisolone 1 g intravenous daily (7 days)  - Therapeutic plasma exchange 1 plasma volume (7 cycles) | 62 | Rituximab |
| 21 | Negative | Negative | ND | ND | None | VA 6/6 both eyes | Normal | ND | No thinning of retinal nerve fiber layer or ganglion cell layer | Methylprednisolone 1 g intravenous daily (5 days) | 2 (until start rituximab) | Rituximab |
| 22 | Negative | ND | Negative | ND | Anti-Beta-2-GP1 IgM 25.42 (<20) | VA 6/6 both eyes | Normal | Normal | No thinning of retinal nerve fiber layer or ganglion cell layer | Methylprednisolone 1 g intravenous daily (5 days) | 1 | Rituximab |
| 23 | Positive | Negative | ND | ND | ANA positive 1:1280 (speckled), anti-dsDNA positive | VA finger count at ½ foot with RAPD positive of the right eye, 6/18 left eye | Sharp discs both eyes with mild pallor of temporal side of the right eye | No delay P100 latency with mildly reduced amplitude both eyes | ND | - Methylprednisolone 1 g intravenous daily (5 days)  - Therapeutic plasma exchange 1 plasma volume (5 cycles) | 14 | Rituximab |
| 24 | Positive | ND | ND | ND | ANA positive 1:160 (fine speckled) | VA 6/6 left eye, hand motion right eye with RAPD positive (same) | Pale optic disc of the right eye | ND | ND | Dexamethasone 4 mg intravenous every 6 hours (3 days) | No corticosteroid tapering | None |
| 25 | Positive | Negative | ND | ND | None | ND | ND | ND | ND | - Methylprednisolone 1 g intravenous daily (3 days)  - Therapeutic plasma exchange 1 plasma volume (7 cycles) | 1 | Rituximab |
| 26 | Positive | Negative | Positive | ND | None | VA 6/6 both eyes | Normal | ND | ND | - Methylprednisolone 1 g intravenous daily (5 days)  - Therapeutic plasma exchange 1 plasma volume (4 cycles)  - Intravenous immunoglobulin 2 g (5 days) | No corticosteroid tapering | Rituximab |

Abbreviations: ANA, antinuclear antibody; anti-beta-2-GP1 IgM, anti-beta2-glycoprotein1 antibody; anti-dsDNA, anti-double stranded DNA; anti-SSA, anti-sjogren's syndrome; AQP4-IgG, aquaporin-4 antibody; CSF, cerebrospinal fluid; MOG-IgG, myelin oligodendrocyte glycoprotein antibody; ND, no data; OCT, ocular coherence tomography; RAPD, relative afferent pupillary defect; TDL, tumefactive demyelinating lesions; VA, visual acuity; VEP, visual evoked potential.

**Supplementary table S4.** Final diagnosis and outcomes of Thai tumefactive demyelinating lesion cohort

| **Case No.** | **TDL: Prognosis** | | | | | | | | | | **Case summary** | |
| --- | --- | --- | --- | --- | --- | --- | --- | --- | --- | --- | --- | --- |
|  | **EDSS at diagnosis** | **EDSS at last follow-up** | **Interval between MRI at diagnosis and follow-up, months** | **Resolution of follow-up MRI** | **CNS-IDD attack after TDL** | | | **Total follow-up time, months** | **Total number of TDL attacks** | **Total number of CNS-IDD attacks** | **CNS-IDD clinical course** | **Final diagnosis** |
|  |  |  |  |  | **Subsequent CNS-IDD attack** | **Time after TDL attack, months** | **Clinical presentations of the attack** |  |  |  |  |  |
| 1 | 2.5 | 0.0 | 2 | Partial | None | - | - | 170 | 1 | 1 | Monophasic | Single attack TDL |
| 2 | 0.0 | 0.0, diagnosed with epilepsy | 2 | Partial | None | - | - | 65 | 1 | 1 | Monophasic | Single attack TDL |
| 3 | 9.5 | 7.5 | 24 | Partial | None | - | - | 48 | 1 | 1 | Monophasic | Single attack TDL |
| 4 | 2.0 | 2.0 | 7.5 | Partial | None | - | - | 26 | 1 | 1 | Monophasic | Single attack TDL |
| 5 | 3.5 | 0.0 | 7.5 | Partial | None | - | - | 70 | 1 | 1 | Monophasic | Single attack TDL |
| 6 | 3.5 | 1.0 | 2 | Partial | None | - | - | 34 | 1 | 1 | Monophasic | Single attack TDL |
| 7 | 3.5 | 0.0 | 4 | Partial | None | - | - | 77 | 1 | 1 | Monophasic | Single attack TDL |
| 8 | 3.5 | 2.0 | 6 | Partial | None | - | - | 104 | 1 | 1 | Monophasic | Single attack TDL |
| 9 | 9.5 | 10.0 (dead) | ND | - | - | - | - | - | 1 | 1 | Monophasic | Single attack TDL |
| 10 | 4.0 | 3.5 | ND | ND | None | - | - | 84 | 1 | 2 | Relapsing-remitting | Single attack TDL with other CNS-IDD attacks |
| 11 | 4.5 | 4.5 | 47 | Partial | None | - | - | 81 | 1 | 1 | Monophasic | Single attack TDL with other CNS-IDD attacks |
| 12 | 4.0 | 4.0 | 1 | Partial | None | - | - | 6 | 1 | 2 | Relapsing-remitting | Single attack TDL with other CNS-IDD attacks |
| 13 | 7.0 | 6.0 | 3.5 | Partial | None | - | - | 12 | 1 | 2 | Relapsing-remitting | Single attack TDL with other CNS-IDD attacks |
| 14 | 8.5 | 8.5 | 3 | Partial | None | - | - | 13 | 1 | 1 | Monophasic | Single attack TDL with other CNS-IDD attacks |
| 15 | 3.0 | 2.5 | 1 | Partial | Present | 7 | Right ON | 48 | 1 | 2 | Relapsing-remitting | Single attack TDL with other CNS-IDD attacks |
| 16 | 8.5 | 3.5 | 6 | Partial | None | - | - | 62 | 1 | 1 | Monophasic | Single attack TDL with other simultaneous CNS-IDD |
| 17 | 5.0 | 5.0 | 0.5 | Partial | None | - | - | 15 | 1 | 1 | Monophasic | Single attack TDL with other simultaneous CNS-IDD |
| 18 | 8.0 | 2.0 | 6 | Partial | Present | 33 | TDL at right fronto-parietal region | 94 | 2 | 2 | Relapsing-remitting | Recurrent TDL |
| 19 | 3.0 | 1.5 | 2 | Partial | Present | 3  14 | - New lesion on follow-up spinal MRI (T8-T10 spinal cord with gadolinium enhancement) (asymptomatic)  - New lesion of follow-up brain MRI (right-sided midbrain and left cerebellum) (asymptomatic) | 17 | 1 | 4 | Relapsing-remitting | Relapsing-remitting multiple sclerosis |
| 20 | 3.0 | 2.5 | 14 | Partial | None | - | - | 49 | 1 | 1 | Relapsing-remitting | Relapsing-remitting multiple sclerosis |
| 21 | 3.5 | 2.0 | 2 | Partial | None | - | - | 20 | 1 | 2 | Relapsing-remitting | Relapsing-remitting multiple sclerosis |
| 22 | 4.5 | 0.0 | 6 | Partial | Present | 6 | TM at T7 level | 12 | 1 | 2 | Relapsing-remitting | Relapsing-remitting multiple sclerosis |
| 23 | 8.0 | 9.5 | 0.5 | Partial | Present | 4  6  11  18 | - Left optic neuritis  - Left optic neuritis with transverse myelitis  - TDL  - TDL | 28 | 3 | 5 | Relapsing-remitting | Neuromyelitis optica spectrum disorder with AQP4-IgG with recurrent TDL |
| 24 | 8.0 | 8.0 | 3 | Partial | Present | 2  37  47 | - Hypothalamic syndrome (hypersomnolence)  - Left ON  - TM | 300 | 1 | 8 | Relapsing-remitting | Neuromyelitis optica spectrum disorder with AQP4-IgG |
| 25 | 9.5 | 8.5 | ND | ND | None | - | - | 6 | 1 | 1 | Monophasic | Neuromyelitis optica spectrum disorder with AQP4-IgG |
| 26 | 8.0 | 6.5 | 3 | Partial | None | - | - | 45 | 1 | 1 | Monophasic | Neuromyelitis optica spectrum disorder with AQP4-IgG |

**Abbreviations:** ADEM, acute disseminated encephalomyelitis; AQP4-IgG, aquaporin-4 antibody; CNS-IDD, central nervous system inflammatory demyelinating diseases; EDSS, expanded disability status scales; LETM, longitudinally extensive transverse myelitis; MRI, magnetic resonance imaging; ND, no data; ON, optic neuritis; TDL, tumefactive demyelinating lesions; TM, transverse myelitis.

**Supplementary table S5.** Definition of neurological presentation domains

| **Neurological presentation domain** | **Definition** |
| --- | --- |
| Motor symptom | Motor symptoms, including weakness (e.g., monoparesis, hemiparesis, triparesis, or quadriparesis), abnormal muscle tone (e.g., spasticity), facial weakness (upper motor neuron), and spastic dysarthria |
| Sensory symptom | Sensory symptoms, including hypoesthesia/anesthesia, hypalgesia/analgesia, hyperesthesia, or allodynia |
| Visual symptom | Visual symptoms, including decreased visual acuity, dyschromatopsia, visual field defects (e.g., scotoma, hemianopia, or quadrantanopia), or visual hallucinations |
| Brainstem syndrome | Focusing on cranial nerve brainstem nuclei involvement, including diplopia, facial hypoesthesia, facial pain, facial weakness (lower motor neuron), oscillopsia, nystagmus, vertigo, sensorineural hearing loss, flaccid dysarthria, dysphagia, dysphonia, intractable hiccup or vomiting |
| Consciousness problem | Focusing on consciousness level, including drowsiness, lethargy, obtunded, stupor, comatose |
| Cognitive problem | Focusing on overt symptoms of each neurocognitive domains including   - Language (e.g., dysphasia, anomia) - Learning and memory (e.g., memory loss) - Perceptual-motor function (e.g., dyspraxia, agnosia) |
| Headache | A pain or discomfort in the head |
| Seizures | Symptoms causing by a sudden, abnormal electrical discharge in the brain (e.g., convulsion) and including non-convulsive seizures |
| Movement disorder | Focusing on   - Abnormal movements (e.g., tremors, dystonia, chorea, athetosis, myoclonus, or tics) - Ataxia (e.g., gait ataxia, limb ataxia) |
| Bowel and bladder dysfunction | Bowel or bladder symptoms, including urinary incontinence, urinary retention, fecal incontinence, or constipation |

**Supplementary table S6.** Brain MRI of the four patients with neuromyelitis optica spectrum disorder with AQP4-IgG

| **Case number** | **T2W FLAIR** | **T1W with gadolinium** |
| --- | --- | --- |
| 23 | 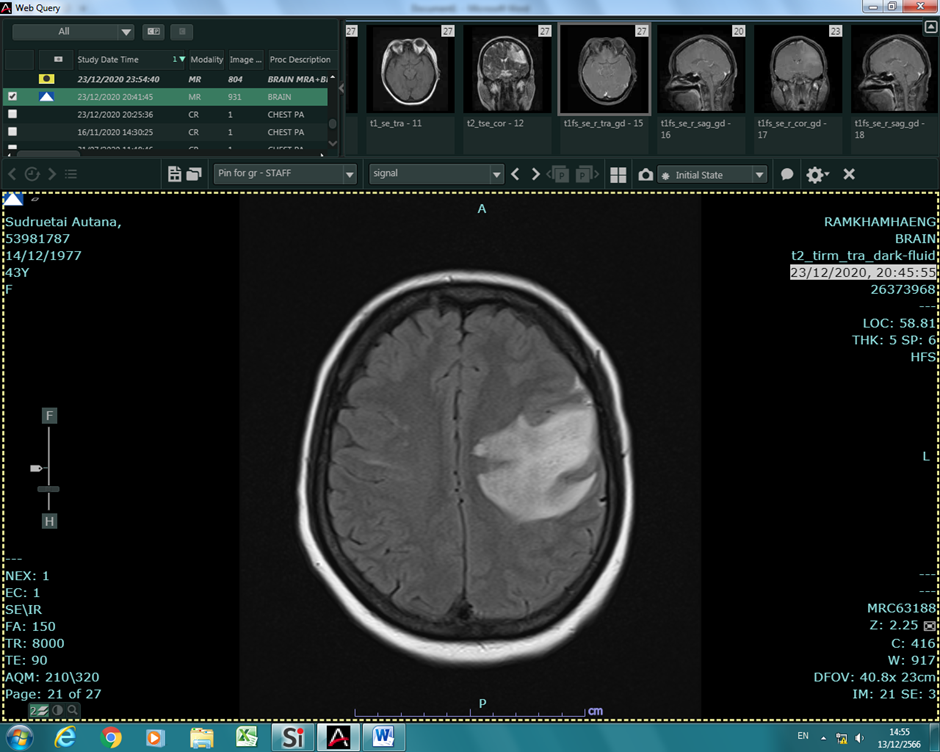 | 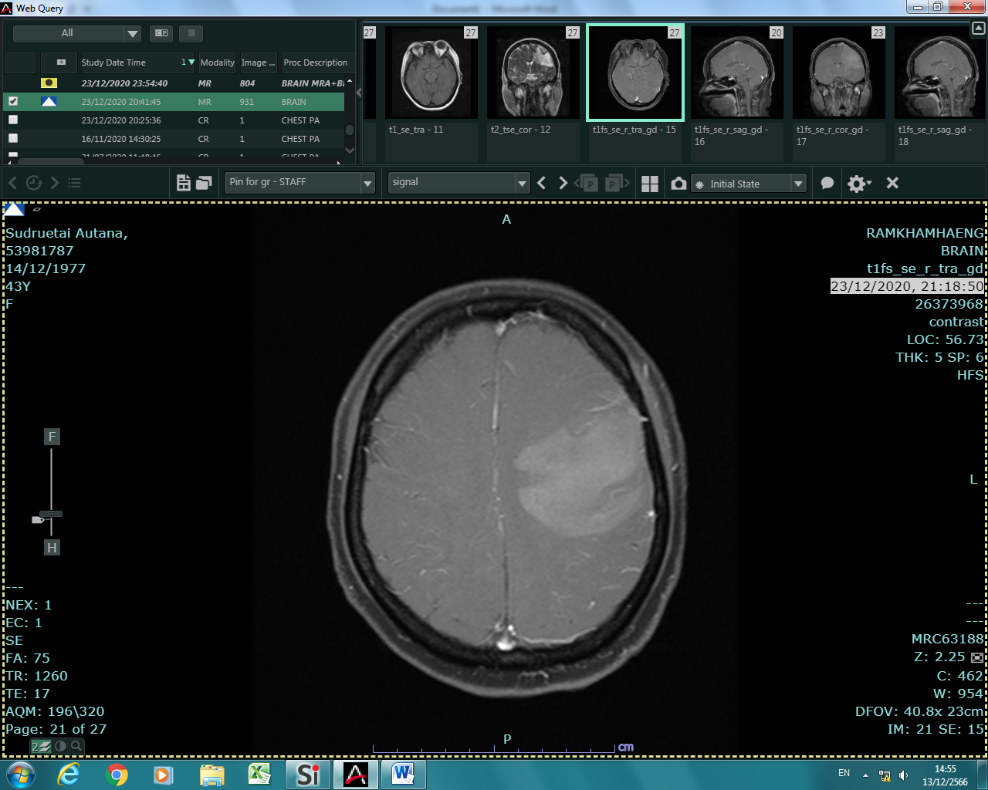 |
| 24 | 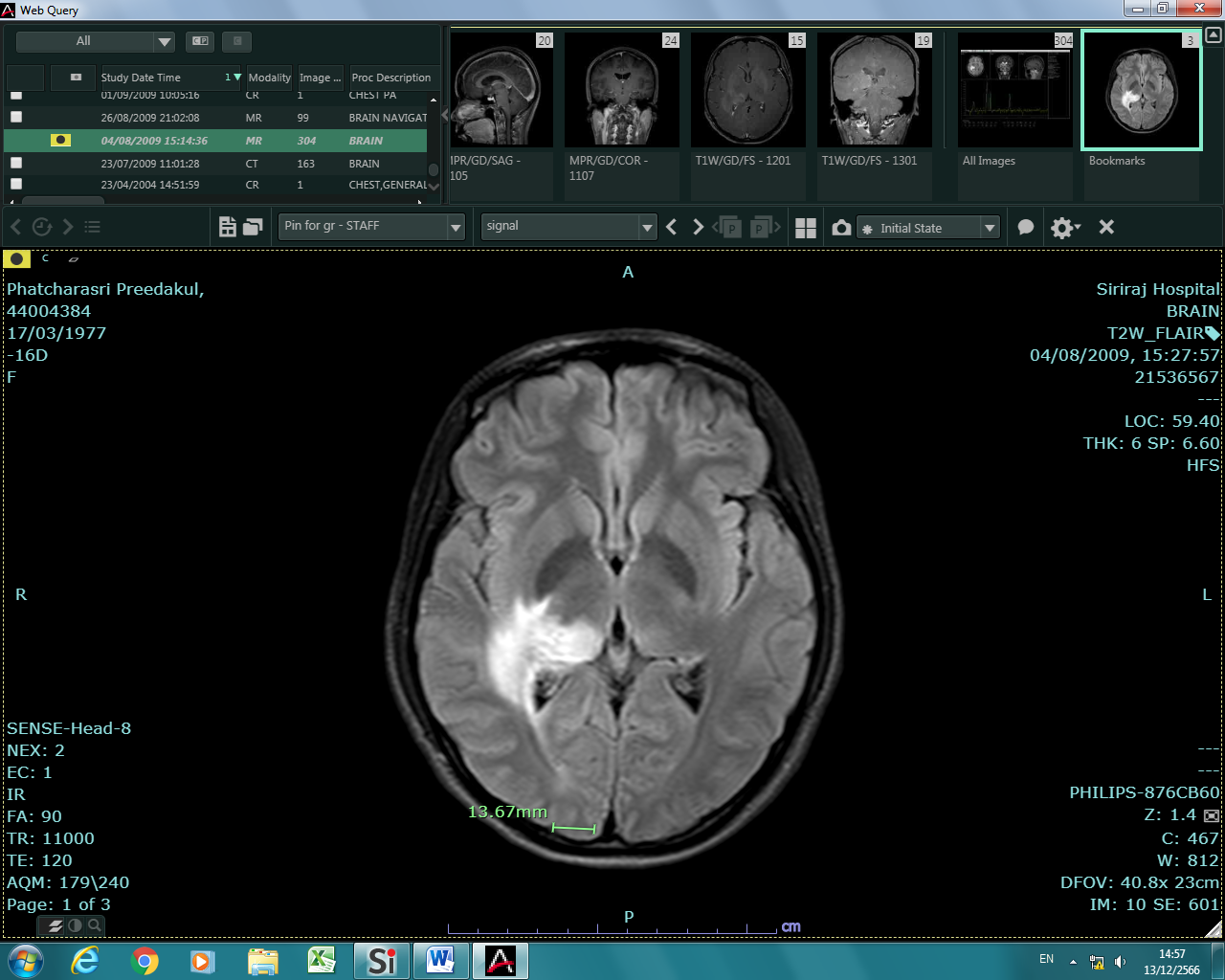 | 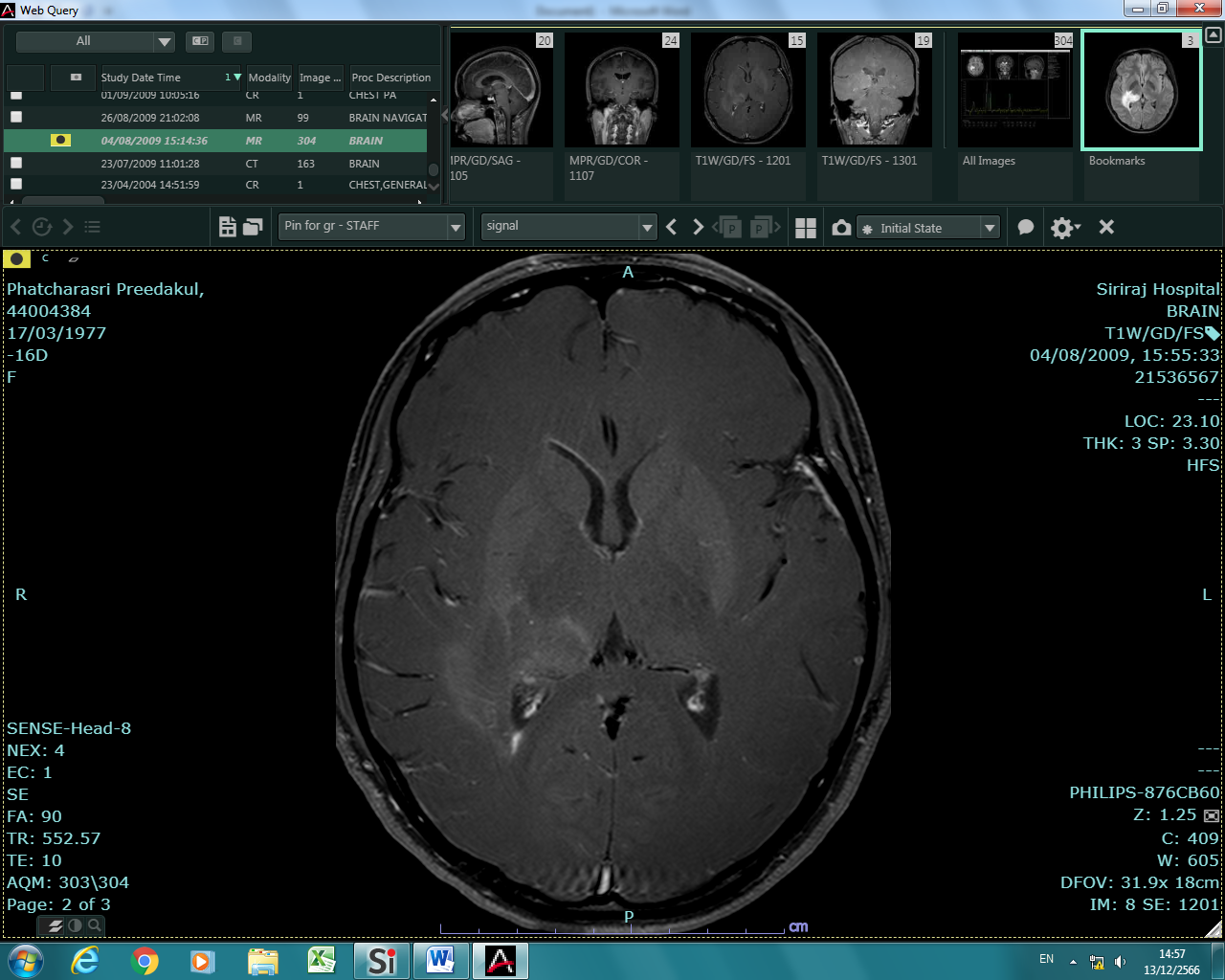 |
| 25 | 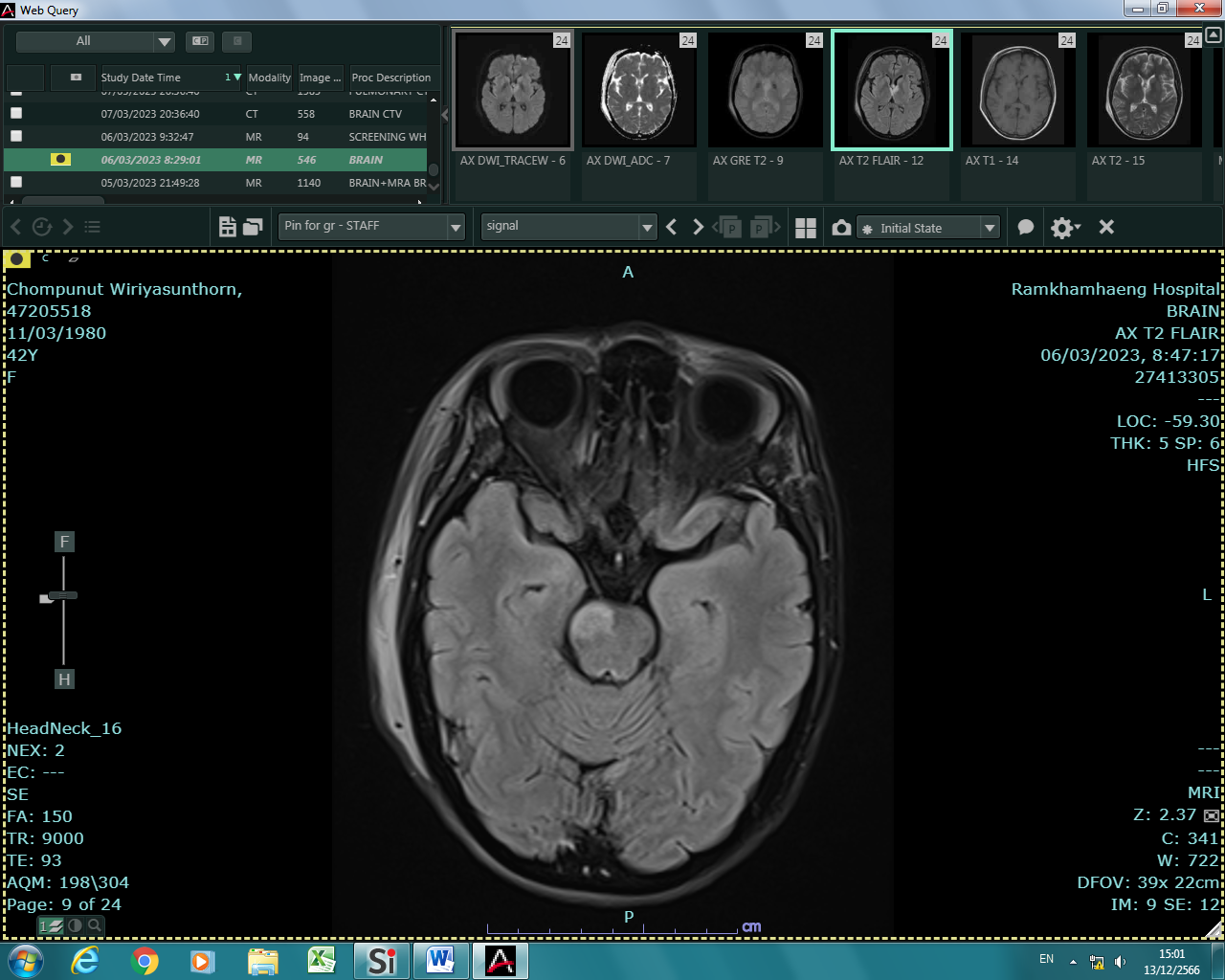 | 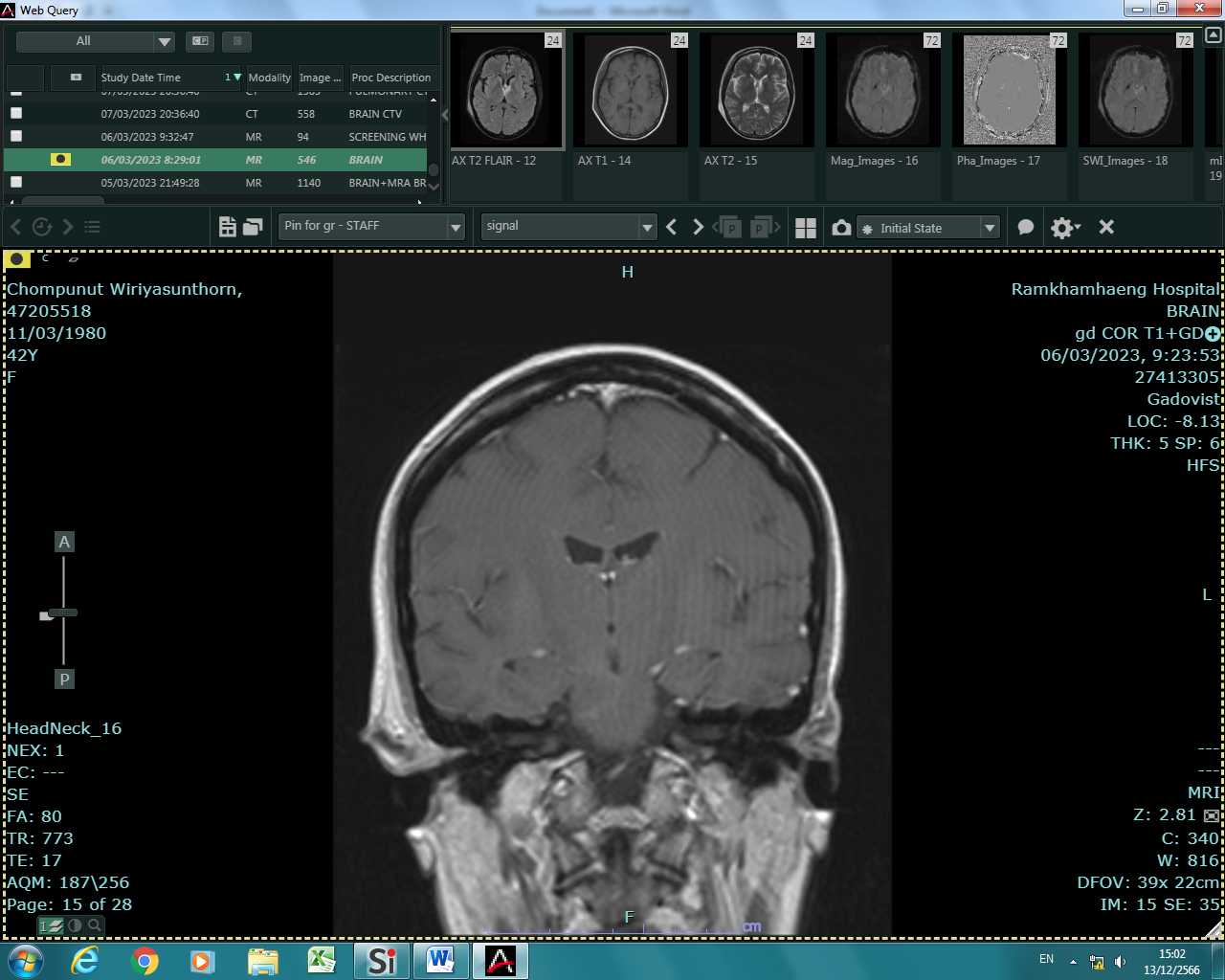 |
| 26 | 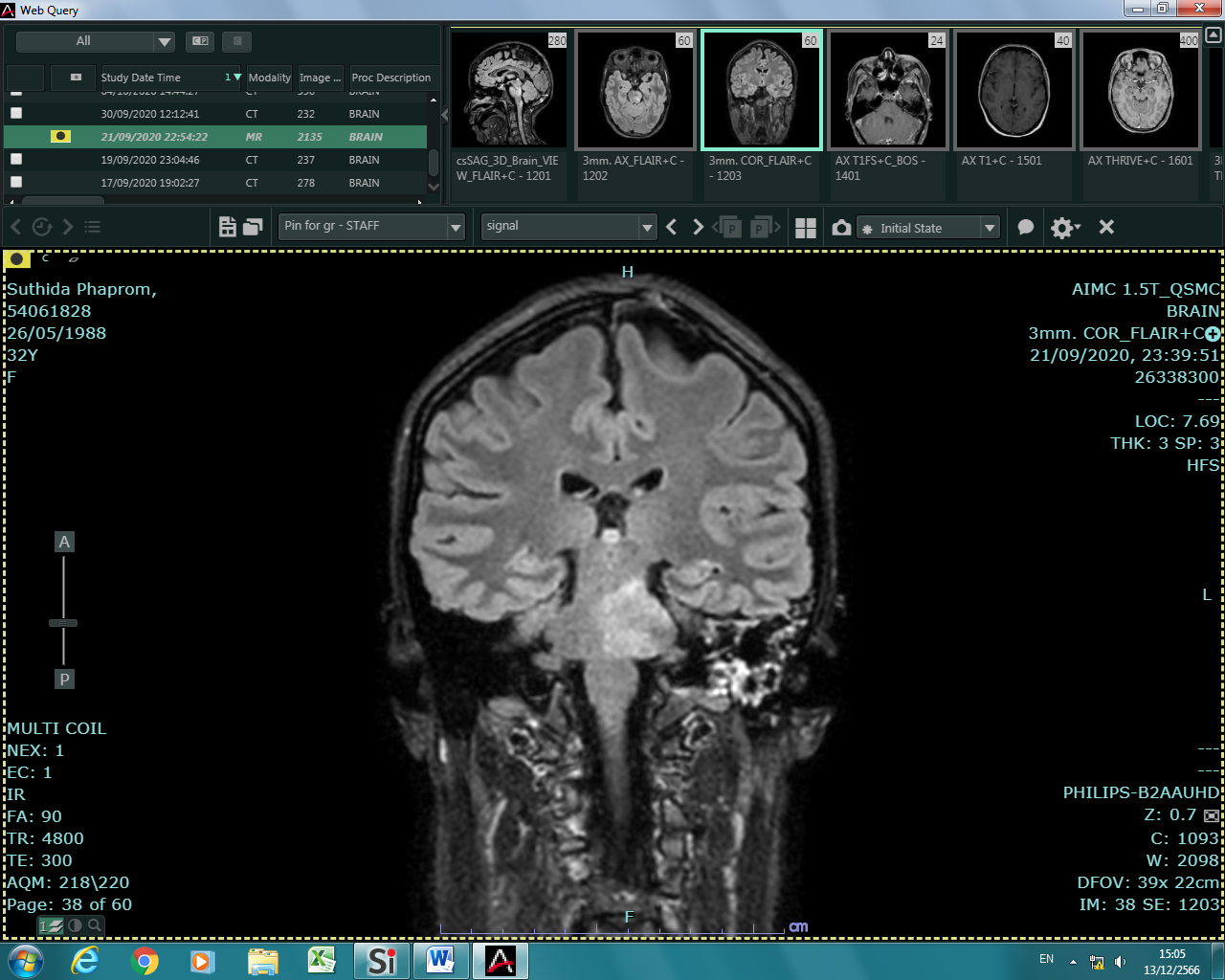 |  |

**Abbreviations:** AQP4-IgG, aquaporin-4 antibody; MRI, magnetic resonance imaging; T1W, T1-weighted imaging; T2W, T2-weighted imaging.
